# Selective enhanced cytotoxicity of amino acid deprivation for cancer therapy using thermozyme functionalized nanocatalyst

**DOI:** 10.1186/s12951-024-02326-6

**Published:** 2024-02-07

**Authors:** Xiuhui Tang, Lijuan Zhang, Mingwang Huang, Fang Wang, Guiqiu Xie, Rui Huo, Renjun Gao

**Affiliations:** 1 Key Laboratory for Molecular Enzymology and Engineering of Ministry of Education, School of Life Sciences, Jilin University, Changchun, 130012 China; 2 School of Pharmaceutical Sciences, Jilin University, Changchun, 130021 China

**Keywords:** Thermozyme drug, Remote regulation, Thermocatalysis-photothermal therapy, Amino acid deprivation, Precise endocytosis, Breast cancer

## Abstract

**Background:**

Enzyme therapy based on differential metabolism of cancer cells has demonstrated promising potential as a treatment strategy. Nevertheless, the therapeutic benefit of reported enzyme drugs is compromised by their uncontrollable activity and weak stability. Additionally, thermozymes with high thermal-stability suffer from low catalytic activity at body temperature, preventing them from functioning independently.

**Results:**

Herein, we have developed a novel thermo-enzymatic regulation strategy for near-infrared (NIR)-triggered precise-catalyzed photothermal treatment of breast cancer. Our strategy enables efficient loading and delivery of thermozymes (newly screened therapeutic enzymes from thermophilic bacteria) via hyaluronic acid (HA)-coupled gold nanorods (GNRs). These nanocatalysts exhibit enhanced cellular endocytosis and rapid enzyme activity enhancement, while also providing biosafety with minimized toxic effects on untargeted sites due to temperature-isolated thermozyme activity. Locally-focused NIR lasers ensure effective activation of thermozymes to promote on-demand amino acid deprivation and photothermal therapy (PTT) of superficial tumors, triggering apoptosis, G1 phase cell cycle arrest, inhibiting migration and invasion, and potentiating photothermal sensitivity of malignancies.

**Conclusions:**

This work establishes a precise, remotely controlled, non-invasive, efficient, and biosafe nanoplatform for accurate enzyme therapy, providing a rationale for promising personalized therapeutic strategies and offering new prospects for high-precision development of enzyme drugs.

**Graphical Abstract:**

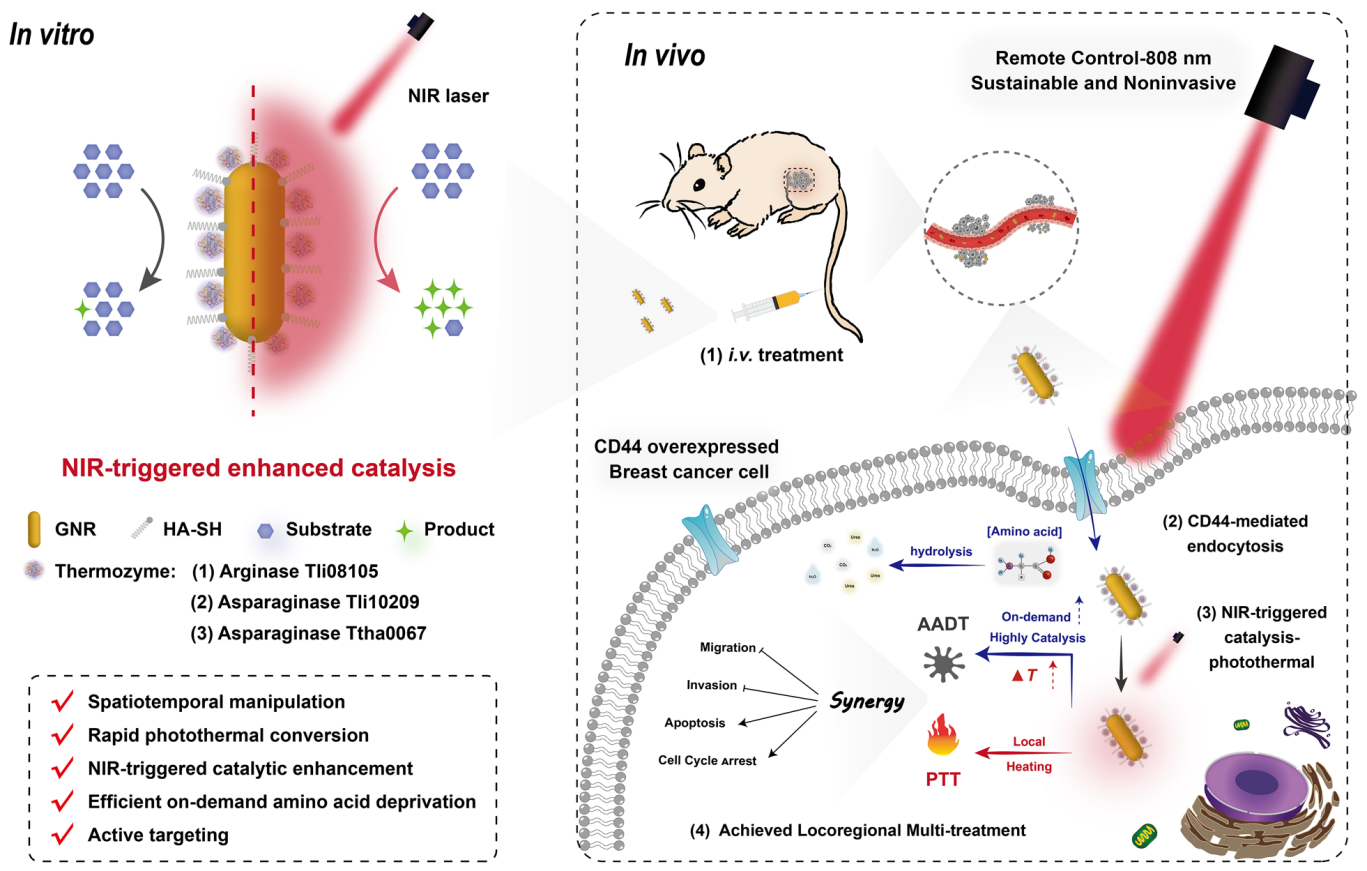

**Supplementary Information:**

The online version contains supplementary material available at 10.1186/s12951-024-02326-6.

## Introduction

Enzyme therapy has emerged as a promising cancer treatment strategy, utilizing specific enzymes to exert catalytic effects in the tumor microenvironment (TME) [1]. Unlike other chemical agents, the substrate specificity and affinity of the enzyme drugs ensure that they do not interfere with other biological processes of the organism, avoiding the cytotoxicity of similar agents on normal cells. However, conventional enzyme therapies, whose efficacy is determined by targeting and activity, are limited by bioavailability to bring significant survival benefit for cancer patients, and conversely, may be associated with adverse effects due to inaccurate dose control [2, 3]. Therefore, actively exploring effective enzyme treatment options that can accurately utilize the catalytic activity of enzymes and have long-term biostability is still an important issue with urgent need.

The extraordinary catalytic ability of enzymes as promising cancer therapeutic tools can be used not only for predrug chemotherapy [4, 5], but also as scavengers to interfere with the intracellular nutrition [6]. Deprivation of essential nutrients, such as amino acids (AAs) and glucose, is a rapid way to directly disrupt and inhibit the differentiation and proliferation of cancer cells. Currently, *E. coli-*derived L-asparaginase, PEGylated arginine deiminase, and human-derived arginase I have been employed in amino acid deprivation therapy (AADT) [7]. Severe nutritional deficiencies produced by enzyme agents can disrupt or cause loss of balance in the tumor microenvironment, triggering S and/or G2/M phase cell cycle arrest, caspase-dependent apoptosis or autophagic cell death pathways [8–10]. The discovered enzyme agents all exert therapeutic catalytic effects at body temperature. Despite the potential applicability and improvements of enzyme therapies in recent years, only a few have been authorized by the FDA [11]. This is presumably owing to the short lifespan and lack of targeting of enzyme drugs in vivo. The low bioavailability means that patients need more frequent treatments, and the resulting immunogenicity and off-target cytotoxicity bring inconvenience and risk [1, 12]. Notably, thermozymes, which have research significance in bioengineering, offer a promising solution. These enzymes have good thermal stability and long half-life, overcoming the biological instability that often occurs in biomedical applications of mesophilic and cryophilic enzymes [13–15], while the low catalytic activity of thermozymes in vivo facilitates avoidance of biological damage to nontarget tissues. These advantages together point to thermozymes as important candidates for valuable drugs [16, 17]. In addition, the ability to modulate the activation of thermozymes into catalytic drugs at specific sites can take advantage of their strengths and break through the weaknesses of conventional enzyme drugs, which are short-acting and performance uncontrollable.

Enzyme regulation is an emerging molecular manipulation method combining nanotechnology with personalized tailoring strategies, which can be used to treat cancer and other diseases by selectively regulating enzymatic activity and effectively reducing adverse effects and side effects in patients. An attractive and practical method of this noninvasive modulation is the optical control method which prevents inactivation due to excessive thermal impacts [18–20]. Metal–organic frameworks [21], polymers [22], gold nanoparticles [23, 24], and other carriers [25] have been developed to enhance the operational stability of biological enzymes and prevent their nonspecific distribution in vivo. Gold nanorods (GNRs) are superior candidates for NIR modulation due to their rapid optical absorption, wavelength-specific localized surface plasmon resonance and photothermal conversion properties [26]. The multivalent coordination capabilities of GNR surfaces can be used for various functional applications, such as targeting [27], sensing [28], and bioimaging [29]. The potential benefits of thermozymes for medical applications have been demonstrated by Liu et al. [23] and Li et al. [30], who investigated the activation of thermophilic acylaminopeptidase and photothermal antimicrobial agents, respectively. Wu et al. created a complementary permeation approach in the NIR-II window to induce papain for penetration of dense tumor mesenchyme [17]. However, precise catalysis of thermozymes as direct drugs to against cancer has not yet been developed with emphasis.

In response to this, we developed a novel thermozyme regulation strategy for precise activation therapy of breast cancer. The near-infrared (NIR) laser was employed as a modulated trigger for thermozyme activation and photothermal therapy (PTT) to achieve locoregional catalysis-photothermal therapy. Incorporating thermozymes (three newly cloned and purified thermophilic arginase/asparaginase from thermophilic bacteria, for AADT) and hyaluronic acid (HA) on GNRs allowed for targeted codelivery, quick enzyme activation, and biosafe therapeutic treatment. HA, a ligand for the CD44 receptor which is overexpressed by almost all cancers [31–33], was chosen as a targeting ingredient. The NIR laser was then used to rapidly catalyze AA hydrolysis, activating the thermozyme activity which is muffled in vivo by temperature limitation. This progressive cumulative in vivo photothermal conversion and AA deprivation created an imbalance in the internal environmental homeostasis of tumor cells and disrupted nutritional balance, leading to inhibited tumor cell proliferation and migration. This biocompatible nanocatalyst, designed on the aforementioned principle and featuring thermozymes as direct drugs rather than an auxiliary factor, is a breakthrough from conventional enzyme therapies. This work establishes a precise, controllable, efficient, and safe nanoplatform for enhanced catalytic therapy, offering new prospects for the development of precision nanocatalytic drugs.

## Experimental

### Synthesis of GHE (GNR@HA@thermozyme) nanocatalysts

The growth of GNRs was executed using the traditional seed-growth method, as previously reported [34]. The following was the method for creating thiol-labelled HA. In a 100 mL HA solution (3 mM), 790.8 mg (4 mmol) of 1-(3-dimethylaminopropyl)-3-ethylcarbodiimide hydrochloride (EDC) and 460.4 mg (4 mmol) N-hydroxysuccinimide (NHS) were added for 2 h of activation. Then, 315.2 mg of cysteine (2 mmol) was mixed and stirred for another 24 h. The product was obtained by lyophilization with a SCIENTZ-12N freeze dryer (Xinzhi Biotechnology Co., LTD, China) after dialysis with a dialysis tube (MWCO = 8000–14,000 Da) for 4 days.

The synthesis of GHE involved the following conjugation: GNRs that had been supercentrifuged to remove excess hexadecyltrimethylazanium bromide (CTAB) surfactant were redispersed in equivalent Milli-Q water and adjusted to pH 9.0 by adding 0.2 M K_2_CO_3_. The GNRs were then conjugated with 0.4 mg/mL thermozyme and 0.1 mg/mL mercapto-HA for 12 h at 4 °C with vigorous stirring. Centrifugation at 12,000 rpm for 10 min was executed to separate the full nanocatalysts from the unconjugated proteins. The residual enzyme concentration in the supernatant was measured utilizing the BCA protein assay. The loading capacity of the thermozyme was thus calculated based on the original concentration and the residue concentration after coupling, and the final loading ratio of thermozyme was 80.0%. According to CTAB precipitation assay [35], the binding efficiency of HA was estimated to be 10.0%.

### Characterization of GHE nanocatalysts

Under an accelerating voltage of 200 kV, the morphology of the nanocatalysts was captured by a HITACHI-H800 microscope (Hitachi, Japan). Using a JXA-840 scanning electron microscope (JEOL, Japan) with an accelerating voltage of 3 kV, scanning electron microscopy (SEM) images of nanocatalysts were captured. Samples were placed on the surface of specimen stubs and coated with platinum while under vacuum. The zeta potential and hydrodynamic size of multiple nanocatalysts was measured by a Nano ZS90 Zetasizer (Malvern, UK). The recording of UV‒Vis absorption spectrum was recorded by a UV2700. The fourier transform infrared spectra (FTIR) was performed using IFS 66v/s spectrometer (Bruker, Germany).

### Detection of catalytic effect of GHE enhanced by NIR irradiation

Briefly, the NIR-activated enzymatic effect of GHE nanocatalyst was achieved under LSR808NL-2 W 808 nm laser-transmitter (Ningbo Yuanming Laser Technology Co., LTD, China) irradiation. Similarly, only NIR irradiation was applied as an auxiliary method during the preheating and the hydrolysis reaction to compare the differences between the NIR-activated thermozyme and the water-bath heating process.

### Utilizability of GHE

The storage stability of the nanocatalysts was periodically tested by storing them at 4 °C for 4 weeks. Biostability was determined by the coexistence of the nanocatalysts with different reagents at 37 °C for a period of time. All experiments took the enzyme activity at the initial placement as 100% to determine the subsequent relative activity.

### Cytotoxicity analysis

MCF7 or MDA-MB-231 cells were inoculated at a density of 5000 cells/well and cultivated overnight in 96-well plates. The various samples prepared were added to Dulbecco’s modified Eagle’s medium (DMEM) containing 10% fetal bovine serum (FBS) and treated cells for 6 h (25 μg/mL thermozyme, 25 μg/mL GE, 25 μg/mL GHE, 20 μg/mL GNR, 20 μg/mL GH (GNR@HA)). Once the medication solution was removed, fresh DMEM (containing 10% FBS) was reintroduced after rinsing with PBS. Each well was irradiated with an 808 nm laser (2.0 W/cm^2^, 5 min) and incubated at 37 °C for 24 h. Another 4 h was spent incubating the medium after adding 20 μL of 5 mg/mL MTT. The suspension solution was carefully removed before measurement, and 150 μL of DMSO was added to each well. The absorbance at 490 nm was detected with a TECAN Infinite F200 Pro microplate metre, and the cell proliferation inhibition efficiency was calculated according to the following formula:

$${\text{Cell viability }}\left( \% \right) = {{{\text{A}}_{{{\text{Sample}}}} } \mathord{\left/ {\vphantom {{{\text{A}}_{{{\text{Sample}}}} } {\text{A}}}} \right. \kern-0pt} {\text{A}}}_{{{\text{control}}}} \times 100\%$$

### Cell apoptosis and cell cycle arrest assay

The subgroup settings and treatments were the same as described above. Propidium iodide (PI) and Annexin V-FITC double-dye labeling were used to investigate cell apoptosis. Using flow cytometry, the double-dye labeling of cells affected by various treatments was fully recorded. PI single staining was used, however, to determine the distribution of the cell cycle. Utilizing flow cytometry to capture the fluorescence generated by the binding of PI to the double-stranded DNA in the cells, the DNA content distribution in the treated cells was measured.

### In vivo synergistic therapy

All the experiments on animals used in compliance following the Institutional Animals Ethics Committee of Jilin University (License Number: 2021SY0719). BALB/c mice, (female, 5 week-old, specific pathogen free (SPF)) were purchased from Beijing Vital River Laboratory Animal Technology Co. Ltd (Beijing, China). Subcutaneous injection of tumor cells was used to create the tumor-bearing BALB/c nude mouse model. After counting and gathering the MCF7 cells in satisfactory growth conditions, the cells were resuspended in sterile saline. Six-week-old nude female mice were subcutaneously injected with cells at a density of 1.0 × 10^6^ into the axilla of the right hind limb. The injection volume of the cell suspension was 100 μL. When the tumor volume reached 80–100 mm^3^, the mice were randomly divided into 6 groups with 6 mice in each group. The mouse model administered by tail vein injections once every three days (5.0 mg/kg thermozyme, 5.0 mg/kg GE, 5.0 mg/kg GHE, 4.0 mg/kg GNR, 4.0 mg/kg GH). Twelve hours after each injection, 2.0 W/cm^2^ NIR laser was continuously irradiated for 5 min at tumor sites. Mouse body weight and tumor size were continuously measured during treatment.

### Pathological evaluations

Following the tumor suppression experiment, the mice were dissected to obtain major organs, including heart, liver, spleen, lung, kidney, and tumor tissues, which were fixed in 4% paraformaldehyde solution for 24 h. After dehydration, paraffin embedding, and tissue section processing, tissue and tumor sections were stained with different dyes and photographed by fluorescence microscopy. Mouse serum was obtained by centrifugation, and the main indicators of renal function and liver function in the serum were measured.

### Statistical analysis

GraphPad Prism 7, Origin 2018, Primer Premier 5, CytExpert, and ImageJ 1.52p were used for all the data collection, processing, and statistical analyses. All data, in both the manuscript and Supplementary Information, are presented as the mean ± standard deviation (SD). Statistical significance of differences between groups was calculated by one-way ANOVA with Tukey’s multiple comparisons test using GraphPad Prism 7 (n.s., not significant; *p < 0.05; **p < 0.01, ***p < 0.001, ****p < 0.0001).

## Results and discussion

### Catalytic-therapeutic mechanism of GHE in breast cancer cell

The high specificity and affinity of enzyme drugs for substrates make them ideal for precision targeted therapies, especially AADT, which was developed based on the differences between normal and malignant cells in terms of energy metabolism [36]. Cancer cells have greater energy demands than normal cells [37] and respond to these demands by increasing the production of transporters, growth hormones, and metabolic enzymes, as well as by promoting angiogenesis to obtain a larger external nutritional supply [2, 38]. In contrast, normal cells possess the ability to synthesize asparagine from aspartate [39], as well as to complete the urea cycle [40]in order to recirculate arginine in response to amino acid deprivation. Additionally, a conserved transcriptional pathway of the integrated stress response (ISR) can help normal cells restore homeostasis under physiological stress [41]. Therefore, by exploiting the higher metabolic dependency and endostatic sensitivity of tumor cells, a potential therapeutic strategy can be used to induce nutritional imbalance in the target tumor tissue, with the expectation of improving therapeutic efficacy while reducing biological damage to normal tissues.

**Scheme 1.**
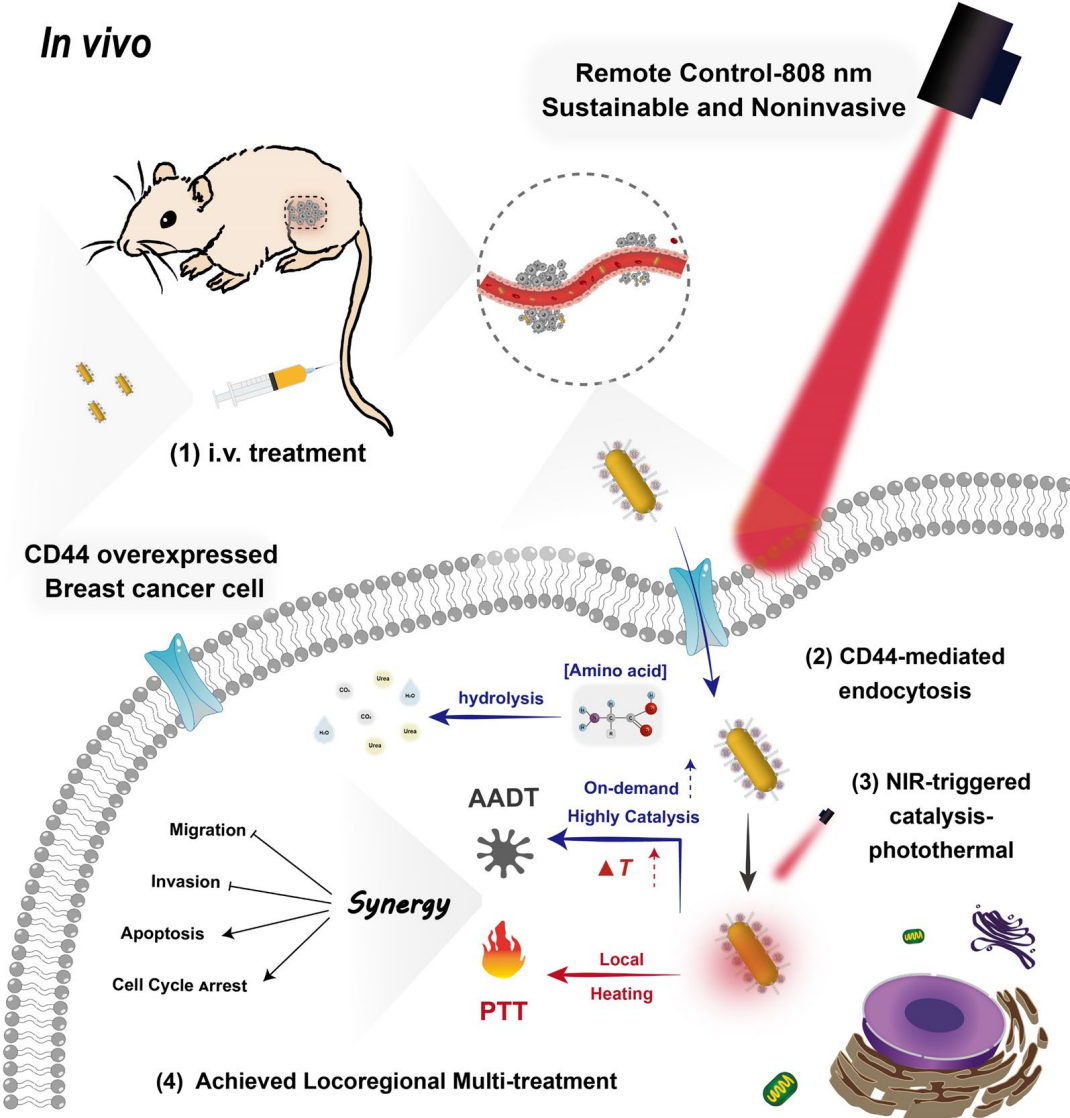
Schematic representation of the action of GHE nanocatalyst for NIR-triggered enhanced locoregional thermoenzymatic-photothermal therapy. Rapid endocytosis of GHE by breast cancer cells is enhanced by active receptor-ligand targeting. Subsequently, a synergistic and precise AA deprivation/photothermal-therapy based on GHE nanocatalyst induces multipath cell death via NIR-triggered procedure

GHE has the potential to maintain modest catalytic activity during the initial phase of in vivo circulation and is predicted to be temperature-limited to avoid accidental amino acid degradation. Moreover, its active targeting ability, when combined with the enhanced penetration and retention (EPR) effect, allows for selective uptake of GHE nanocatalyst by MCF7 and MDA-MB-231 cells overexpressing CD44 [42, 43]. When locoregionally exposed to NIR laser (808 nm) radiation, the local heat generated by GNRs and the internal energy of molecular oscillations activate thermozymes [44], resulting in intracellular amino acid deficiency and endostatic imbalance, which can lead to tumor cell death. The nanocatalysts based on the novel optical-catalytic concept may be able to maximize therapeutic potential by progressively enhancing the cumulative synergistic photothermal [45] and catalytic performance **(**Scheme 1**)**.

### Newly screened thermophilic AA hydrolases

**Fig. 1:**
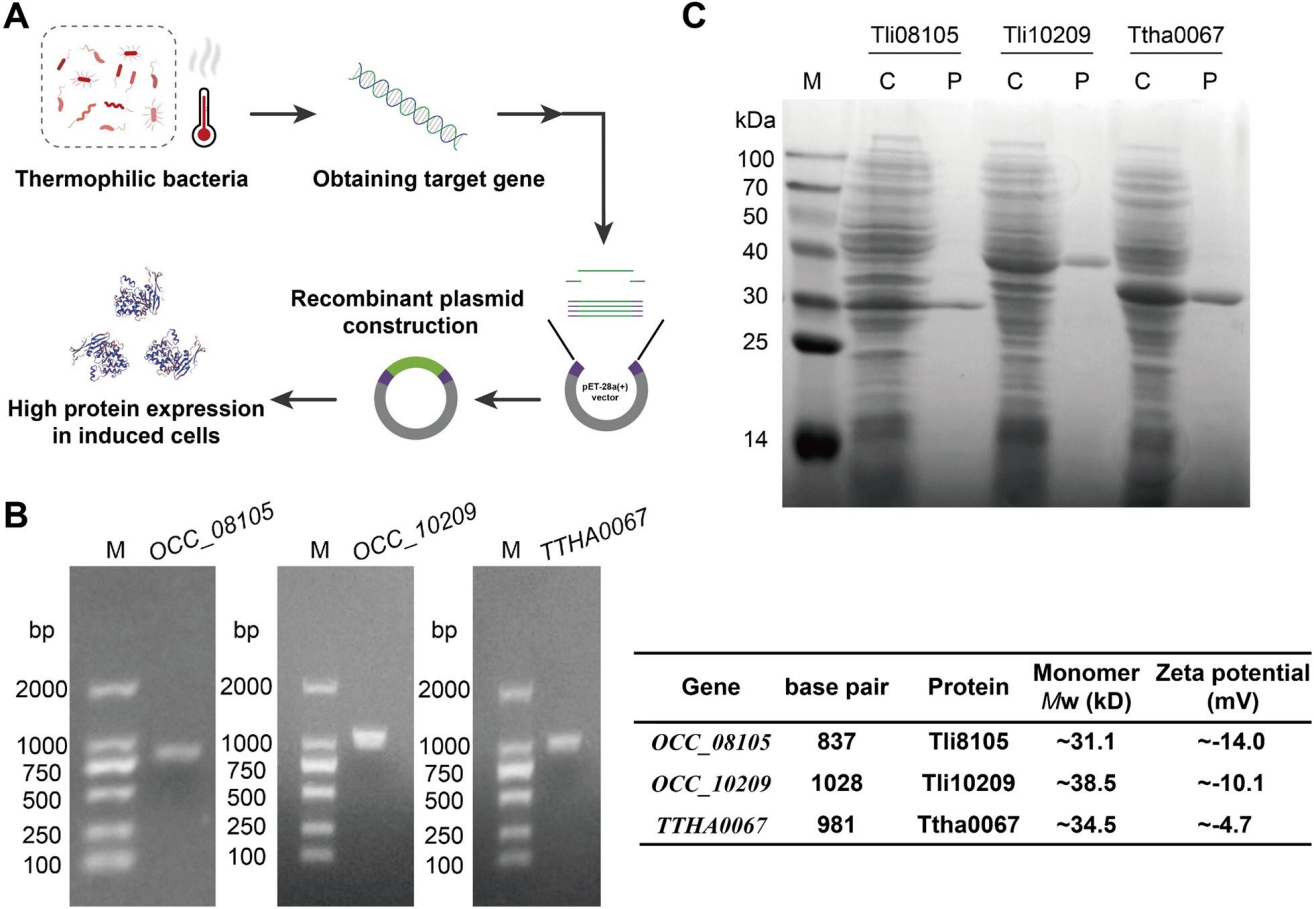
Cloning and purification of thermozymes from thermophilic bacteria.** A** Schematic diagram of the application of recombinant DNA for the protein obtaining from thermophilic bacteria. **B** PCR amplification of target gene fragments by agarose gel electrophoresis. M: DL2000 DNA marker. Gene length, protein monomer molecular weight and zeta potential are presented in the table (right). **C** Coomassie brilliant blue staining of proteins following SDS-PAGE. M: molecular weight markers with size indicated in kDa; C: crude extracts of recombinant proteins; P: purified proteins by Ni^2+^-NTA chromatography

Bacterial enzymes from thermophilic or harsh environment strains demonstrate significant stability [14]. The genetic recombination technique **(**Fig. 1A**)** was utilized to identify, clone and express three AA hydrolases from the genome sequence of thermophilic bacteria deposited in GenBank (Additional file 1: Table S1). The target gene fragments **(**Fig. 1B**)** and the expression of recombinant proteins **(**Fig. 1C**)** of arginase Tli08105, as well as asparaginase Tli10209 and Ttha0067, were validated, as described in the Additional file 1.

### Enzymatic properties of thermozymes

The effect of temperature (30–90 °C) on the hydrolysis of AAs (pH 7.0) was determined to determine the optimal temperature for hydrolyzing L-arginine by Tli08105; this was determined to be 70 °C. Similarly, thermophilic asparaginases Tli10209 and Ttha0067 also exhibited a strong catalytic contrast at low- and high-temperature comparisons (Additional file 1: Figs. S1(A–C). This optimal temperature surpasses that of conventional enzyme preparations, suggesting the possibility of enzyme tunability **(**Tables 1, 2**)**. Additionally, the three thermozymes were found to have the highest hydrolytic activity in slightly alkaline solutions. Tli10209 exhibited a relative optimum activity (pH 9.0) of 87% within pH 6.0–12.0, while Tli08105 had a relative optimum of 80% (pH 10.0) within pH 3.0–12.0 (Additional file 1: Figs. S1(G–I). These results imply a stronger applicability in microacidic systems, similar to the tumor cell microenvironment [46].

**Table 1:** Properties of arginases from different resources

Resources	Temperature (°C)	pH	K_m_ (mM)	*V*_max_ (μmol/min/mL)	*k*_cat_ (s^−1^)	*k*_*cat*_*/K*_*m*_ (mM^−1^ s^−1^)	References
Human	40	10.0	4.9	*NR*	460	93.9	[49]
Rat liver	40	9.8	1.0	*NR*	2600	2600	[50]
Camel liver	70	9.0	7.1	*NR*	*NR*	*NR*	[51]
*Sulfobacillus acidophilus*	70	7.5	34.4	*NR*	458.3	13.3	[52]
*Bacillus caldovelox*	60	9.0	3.4	*NR*	*NR*	*NR*	[53]
*Thermococcus litoralis*	70	10.0	1.059	0.9612	1.247	1.35	This work

*NR* not reported

**Table 2:** Properties of asparaginases from different resources

Resources	Temperature (°C)	pH	*K*_m_ (mM)	*V*_max_ (μmol/min/mL)	*k*_cat_ (s^−1^)	*k*_*cat*_*/K*_*m*_ (mM^−1^ s^−1^)	References
*Escherichia coli*	37	7.0–8.0	0.015	*NR*	24	1600	[54, 55]
*Erwinia chrysanthemi*	45	7.5	0.058	*NR*	23.8 × 10^3^	411.8 × 10^3^	[55, 56]
*Thermococcus litoralis*	90	9.0	4.340	323.9	2077.19	478.68	This work
*Thermus thermophilus* HB8	80	9.0	6.178	210.9	1213.52	196.42	This work

*NR* not reported

The thermozymes also exhibited good tolerance to medium–high temperature (Additional file 1: Figs. S1(D–F) and pH (Additional file 1: Figs. S1(J–L), with Tli08105 and Ttha0067 retaining 67.35% and 61.90% of their initial viability, respectively, after 72 h of storage at 80 °C. This is beneficial for reducing production costs and optimizing production efficiency compared to clinically used normothermic enzyme preparations [11, 13].

The kinetic parameters of the thermozymes were calculated using the Lineweaver‒Burk plot of the Michaelis‒Menten equation at different concentrations of L-arginine/L-asparagine. The Km value of L-Arg hydrolyzed by Tli08105 at 70 ℃ (1.06 mM) revealed a greater substrate affinity than human-derived arginase (Table 1). Studies have also detailed the millimolar level *K*_m_ values of bacteria-derived asparaginases, including thermophilic asparaginases [11] (Table 2). Considering the physiological concentrations of Arg (80–100 μM) [47] and Asn (~ 50 μM) [48] in adult blood, the *K*_m_ of the therapeutic enzyme needs to be low micromolar range to be clinical relevant. Thus, the basic enzymology properties of the thermozymes are consistent with the requirements of the enzyme preparation for this study model.

### Preparation and characterization of GHE nanocatalyst

**Fig. 2:**
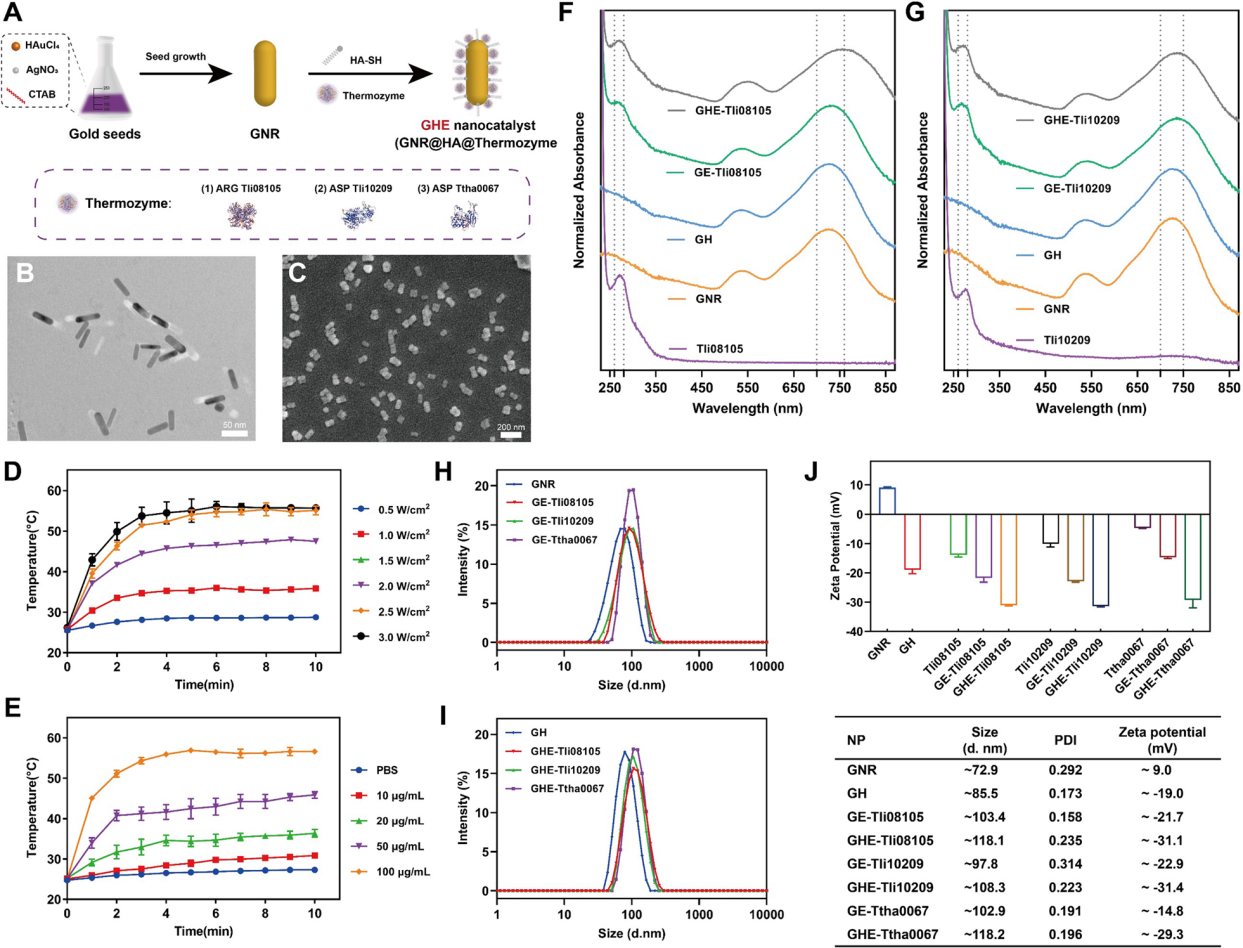
Synthesis and characterization of GHE nanocatalyst. **A** Schematic representation of the synthesis procedure of GHE nanocatalyst. **B** TEM image of GNRs. Scale bar: 50 nm. **C** SEM image of GHE-Tli08105. Scale bar: 200 nm. Evaluation of thermogenesis of aqueous GNRs mediated by 808 nm NIR **D** with 50 μg/mL at different power densities; **E** with different concentrations at the density of 2.0 W/cm^2^. UV–Vis absorption spectrum of **F** arginase Tli08105 and **G** asparaginase Tli10209 before and after assembly with GNR or GH. The hydrodynamic size of **H** GNR- and **I** GH-thermozyme nanoparticles by DLS. **J** Zeta-potential of GNRs and GH before and after thermozyme-modification. The particle size, polymer dispersity index (PDI) and zeta-potential are presented in the table (bottom)

Generic nano-rod structures of GNRs were synthesized according to the classical seed-mediated growth method [34], as observed from the Transmission Electron Microscopy (TEM) in Fig. 2B, with an aspect ratio of 3.5 (44.0 × 12.6 nm). To characterize the photothermal conversion effect of GNRs, an 808 nm NIR laser emitter was utilized. The photothermal conversion within 0–1 min indicated a high nonradiative leap efficiency, which positively contributed to PTT. Additionally, the temperature stability was maintained after the thermal limit (57 ℃) was reached within 5 min, confirming the excellent photothermal stability (Fig. 2E). This result proved that GNRs can be rapidly triggered by NIR to reach the desired therapeutic temperature, while their photothermal characteristics remain unaltered by enzyme coupling [57]. Furthermore, the power parameter of 2.0 W/cm^2^ was chosen to consider the laser power tolerance threshold of human tissue skin with the reported treatment modalities (Fig. 2D) [58].

The enable generic and efficient targeting strategies, integrating components that differentiate specific molecular differences between cancerous and healthy cells is essential [59]. Au–S covalent bond cooperation is a general strategy for a variety of gold nanoparticles [26] that facilitates the spontaneous construction of GHEs and prevents the loss of enzymatic activity in multistep operations. Figure 2A presents a maneuverable strategy for this purpose. To minimize the incidence of intrinsic enzyme aggregation, which is a common physical stabilizing feature of CLEA during initial immobilization, simultaneous enzyme coupling in the presence of sulfhydrylated HA was intended [60, 61]. The HA-Cys conjugate was obtained by covalently cross-linking the carboxyl group of HA with the primary amine group of Cys by EDC/NHS chemistry. Peaks at 1645 cm^−1^ and 1417 cm^−1^ were attributed to the asymmetric bending and symmetric stretching peaks of the carboxyl group in HA. The FTIR results showed the sulfhydryl peak at 2549 cm^−1^ after binding Cys, confirming the successful synthesis of the HA-Cys conjugate[62, 63]. The disappearance of the sulfhydryl absorption peak after modification of GNR confirmed the successful immobilization of GNR-HA (Additional file 1: Fig. S4). This immobilization mode also improved the relative water solubility of nanoparticles. The recovery activity of enzymes and the homogeneity of the nanocatalysts were guaranteed by coupling thermozyme and HA-SH at doses of 0.4 mg/mL and 0.1 mg/mL, respectively. Additionally, the nanocatalyst is biocompatible due to the high chemical compatibility between GNRs, natural proteins, and glycosaminoglycans; as well as the absence of toxic chemical reagents during the conjugation process, ensuring its biosafety in vivo.

Notably, three thermozymes were studied as NIR-triggered GHE nanocatalysts (Fig. 2A). The nanocatalysts were characterized by SEM (Fig. 2C and Additional file 1: Fig. S5), hydrodynamic size, zeta-potential and UV‒Vis spectroscopy. The representative SEM image of GHE-Tli08105 revealed no distinct morphological alternation and with a retained rod-shaped (Fig. 2C). Due to the large pores of the tumor vasculature (sometimes several hundred nm to 2 μm) [64], GE/GHE nanocatalysts favoured EPR and reduced renal filtration (Fig. 2H, I). In addition to molecular size, physicochemical characteristics such as isoelectric point also play a role in renal filtration. Ionization of the protein side chains, which leads to free charge on the GNR surface, occurs concurrently with enzymatic coupling. After modification by HA and thermozyme, the zeta-potential of GHE tends to be negatively charged **(**Fig. 2J**)**. Anionic nanomaterials typically have longer circulating half-lives due to their electrostatic barrier of glomerular filters created by negatively charged glycosaminoglycans [65]. The successful coupling was further evidenced by the change in potential values, with an increase in absolute value implying nanocatalyst stability. Furthermore, enzyme coupling influences the refractive index of the medium surrounding GNRs, accompanied by a change in the dielectric constant that may directly affect the LSPR effect [26]. The longitudinal UV absorption peak of GNRs shifted slightly from 721 nm to 736–758 nm with the attachment of thermozymes and/or HA, while the transverse peak remained relatively unchanged (Fig. 2F, G and Additional file 1: Fig. S6). The larger absorption peak inferred a photothermal responsiveness of the NIR trigger. Further evidence of the successful coupling of enzymes was provided by the characteristic absorption peak of the protein at 280 nm. The analysis mentioned above demonstrated that GHE was successfully built.

### NIR laser triggered the enzymatic activities of GHE nanocatalyst

**Fig. 3:**
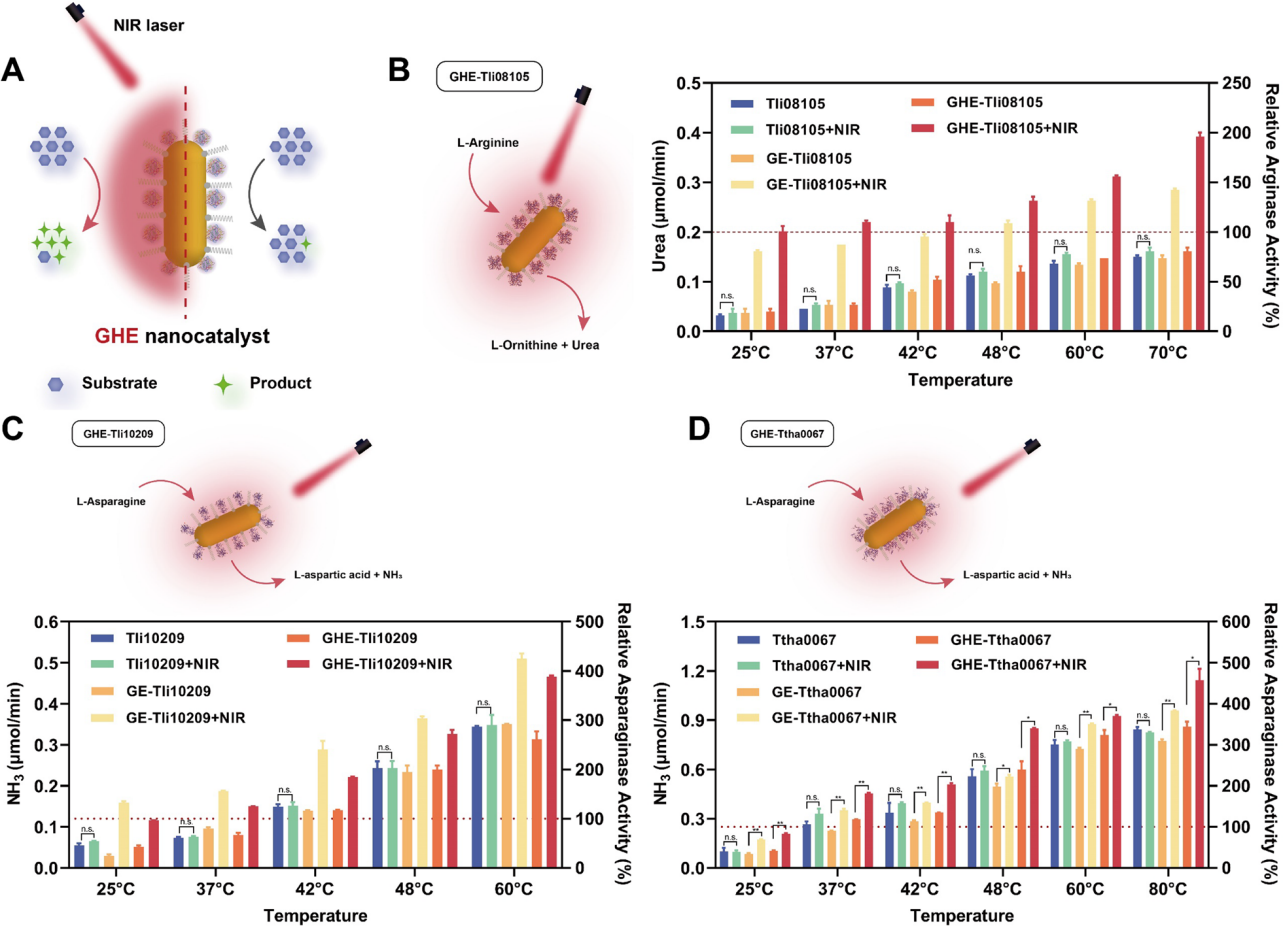
NIR laser triggered the enzymatic activities of GHE nanocatalyst. **A** Thermozyme-based GHE nanocatalyst showed enhanced enzymatic activity by light irradiation (left panel) compared to non-irradiation (right panel). Schematic of the hydrolysis of respective amino acids catalyzed by three GHEs upon NIR irradiation. Evaluation of the enzymatic activity of GHE based on **B** arginase Tli08105, **C** asparaginase Tli10209 or **D** asparaginase Ttha0067 in the presence or absence of 808 nm NIR irradiation at 2.0 W/cm^2^. The bulk temperature in solution was maintained at different gradient. Data are shown as the mean ± SD (n = 3); n.s. represents nonsignificant difference, and **p < 0.01, *p < 0.05 (one-way ANOVA with Tukey’s multiple comparisons test)

Further studies revealed that irradiation assistance can greatly enhance GHE catalysis at various temperatures (Fig. 3). NIR markedly activated GHE-Tli08105 and GE-Tli08105, promoting L-arginine hydrolysis to 4.1 and 3.3 times that of the unaided state, respectively, when the nanosystem was kept at 37 °C (Fig. 3B). Similarly, the hydrolysis of L-asparagine catalysed by GHE-Tli10209 and GE-Tli10209 increased by 1.9 and 2.0 times at 37 °C, respectively (Fig. 3C). Both GE-Ttha0067 and GHE-Ttha0067 catalyzed hydrolysis increased by 1.5-fold at 37 °C (Fig. 3D). This suggested that the close binding of GNR and thermozyme can induce enhanced enzymatic activity, possibly because covalent coupling shortens the physical distance, while making the photothermal transfer and dominant conformational transition more direct and rapid. Surprisingly, the high-temperature catalytic impact of Tli08105 (70 °C) was surpassed by the NIR-activated GHE-Tli08105 activity (37 °C). It has been suggested that the intraregional energy of local molecular oscillations is also significant in complement to directed photothermal transmission [44]. The thermophilic properties in this work complement NIR-mediated regulation perfectly, as local warming of the microenvironment is more conducive to the protection of enzyme conformational integrity and extending the enzyme lifetime than macroscopic heating [44].

Nanoparticle stability is essential for biomedical applications. In intricate biological fluids, nanodrugs tend to cluster and are eventually cleared by the reticuloendothelial system (RES), ultimately reducing effectiveness [64]. To ascertain the impact of various media on its stability, we measured the catalytic activity and the hydrodynamic size of nanocatalysts in cell culture medium, PBS buffer, and hyaluronidase-containing solution for 24 h (Additional file 1: Fig. S7 and S8). The biological stability and dispersion of GHE may contribute to the prolonged circulation time, thus bypassing the clearance mechanism and exerting enhanced anticancer efficacy. Additionally, the storage stability of GE/GHE at 4 ℃ for 28 days was also demonstrated (Additional file 1: Fig. S9). The analysis revealed that Au–S bonds formed by individual cysteines exposed to protein are positively significant in both protecting Cys and sustaining disulfide bonds. The spatial maintenance of the overall structure of the protein facilitates the enhancement of enzyme stability and prolongation of service life, giving GHE potential as biopharmaceutical.

### NIR irradiation enhanced the anticancer activity in vitro

**Fig. 4:**
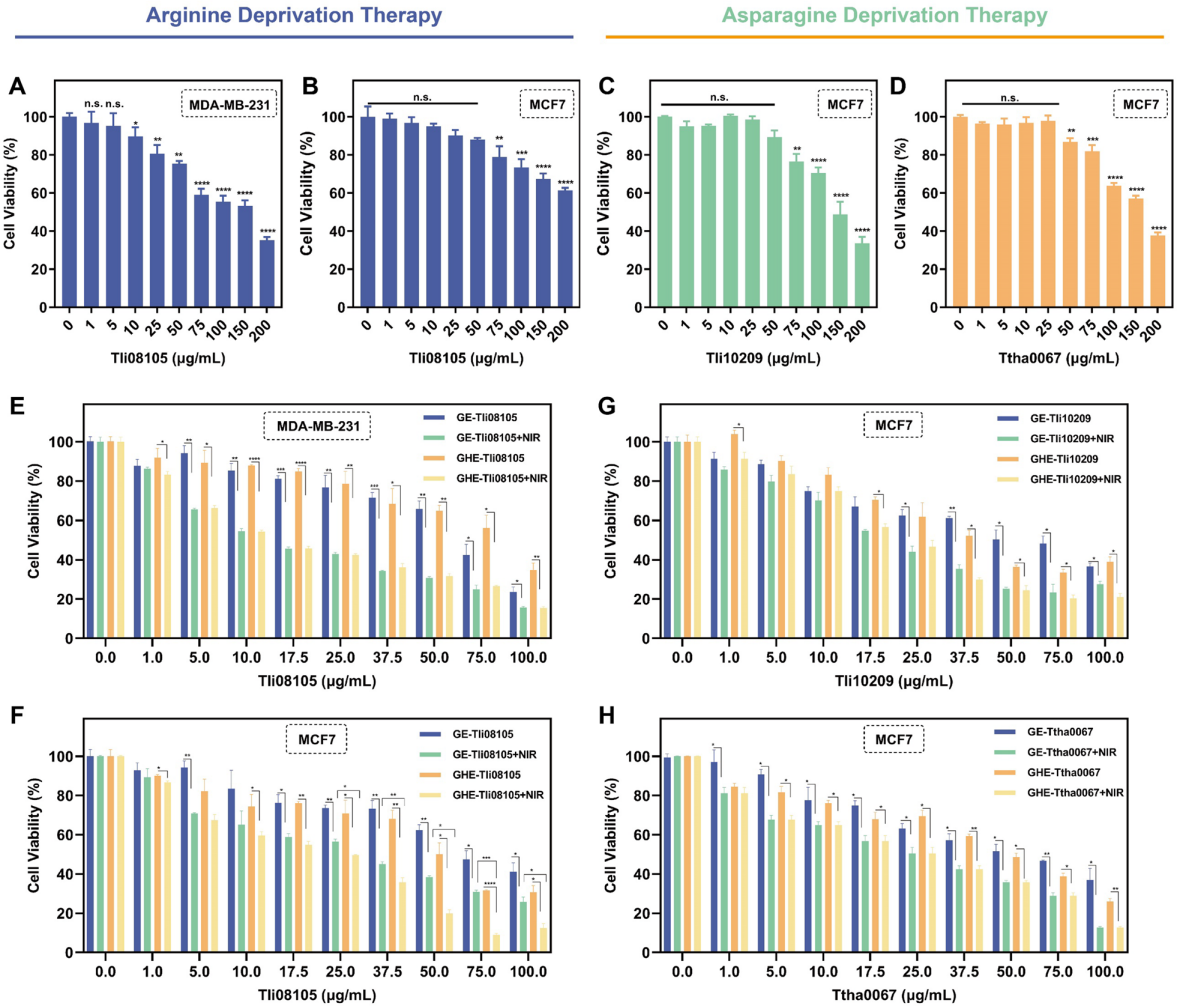
NIR irradiation enhanced the anticancer activity of thermocatalysis by GHE nanocatalyst. Relative viability of **A** MDA-MB-231 and **B** MCF7 cells incubated with various concentration of Tli08105. Relative viability of MCF7 cells incubated with various concentration of **C** Tli10209 and **D** Ttha0067. The viability of **E** MDA-MB-231 and **F** MCF7 cells treated with different concentration of GE-/GHE-Tli08105 nanocatalysts at the absence and presence of NIR laser irradiation (2.0 W/cm^2^ for 5 min). The viability of MCF7 cells treated with different concentration of **G** GE-/GHE-Tli10209 and **H** GE-/GHE-Ttha0067 nanocatalysts with/without NIR laser irradiation (2.0 W/cm^2^ for 5 min). Data are shown as the mean ± SD (n = 3). (*p < 0.05; **p < 0.01, ***p < 0.001, ****p < 0.0001)

To investigate the cytotoxicity parameters and the anticancer activity of different dosages of thermozymes, the viability of human breast cancer cells was determined via MTT assay (Fig. 4(A–D). Two breast cancer cell lines were chosen as models to test the sensitivity to thermophilic arginase treatment. Differences in the expression of key enzymes in the urea cycle affect the self-synthesis of arginine in TME. Yu et al. demonstrated that human arginase 1 had strong cytotoxic to MCF7 cells even though they were argininosuccinate synthetase (ASS) positive [65]. Qiu et al. found a higher relative sensitivity of arginine deiminase (ADI)-PEG20-induced proliferation inhibition in MDA-MB-231 cells compared to MCF7, given their lower abundance of ASS 1 mRNA and ASS 1 protein [66]. Wang et al. proved MDA-MB-231 cells that were also ornithine transcarboxylase (OTC)-negative [67]. OTC and ASS, key enzymes for arginine synthesis from citrulline, facilitate pretreatment screening as valid predictive markers of arginase therapeutic responsiveness. MCF7 cells showed slight resistance to Tli08105 treatment compared to treated MDA-MB-231 cells (Fig. 4A). Tli08105 (225.5 U/mg) had an anti-proliferative effect comparable to the *Bacillus caldovelox* arginase in MCF7 cells [68] (Fig. 4B). Likewise, the lower activity under physiological conditions limits further application in cancer treatment. Photothermal triggering of the thermophilic arginase catalysis in TME was used to balance its thermo-stability and anticancer activity. Activation of GE/GHE-Tli08105 nanocatalysts by locoregional NIR laser effectively inhibited cell proliferation, with GHE-Tli08105 + NIR (25 μg/mL) treatment of MDA-MB-231 and MCF7 cells retaining only 42.5% and 49.6% cell viability, respectively (Fig. 4E, F). This indicates the damage caused by NIR activation catalysis.

Invasive breast cancer cells with mesenchymal features exhibit more nutritional asparagine and glutamine dependence compared to less invasive epithelial cells [69]. The concentration-dependent carcinogenic effect of thermozymes was evident in the dose‒response curve. Comparing commercial asparaginase (clinical enzyme preparation), thermophilic asparaginase Tli10209 (11.6 U/mg) and Ttha0067 (42.78 U/mg) treatments at relatively high doses demonstrated similar inhibitory effects [70] (Fig. 4C, D). Low catalysis during in vitro circulation of the wild-type bacterial enzymes was shown to avoid the off-target toxicity and ensure biosafety. Comparison of the irrational strong activity and unintended catalysis of commercially normothermic PEGylated enzymes leading to low survival in HCC clinical trials showed the potential of thermozymes [2, 7]. GHE-Tli10209 + NIR (25 μg/mL) and GHE-Ttha0067 + NIR (25 μg/mL) treatment of MCF7 cells retained 46.8% and 50.4% of cell viability, respectively (Fig. 4G, H). Using human normal mammary epithelial cell line MCF10A as a model, no obvious cytotoxicity was observed for nanocatalyst with cell viability of > 90% in the selected concentration (0–100 μg/mL) (Additional file 1: Fig. S10).This demonstrates the sensitivity treatment between Arg-/Asn-deprivation and photothermal enhancement, reinforcing the notion of synergistic cancer therapy rather than single agents.

### In vitro anticancer performance of AADT-PTT

**Fig. 5:**
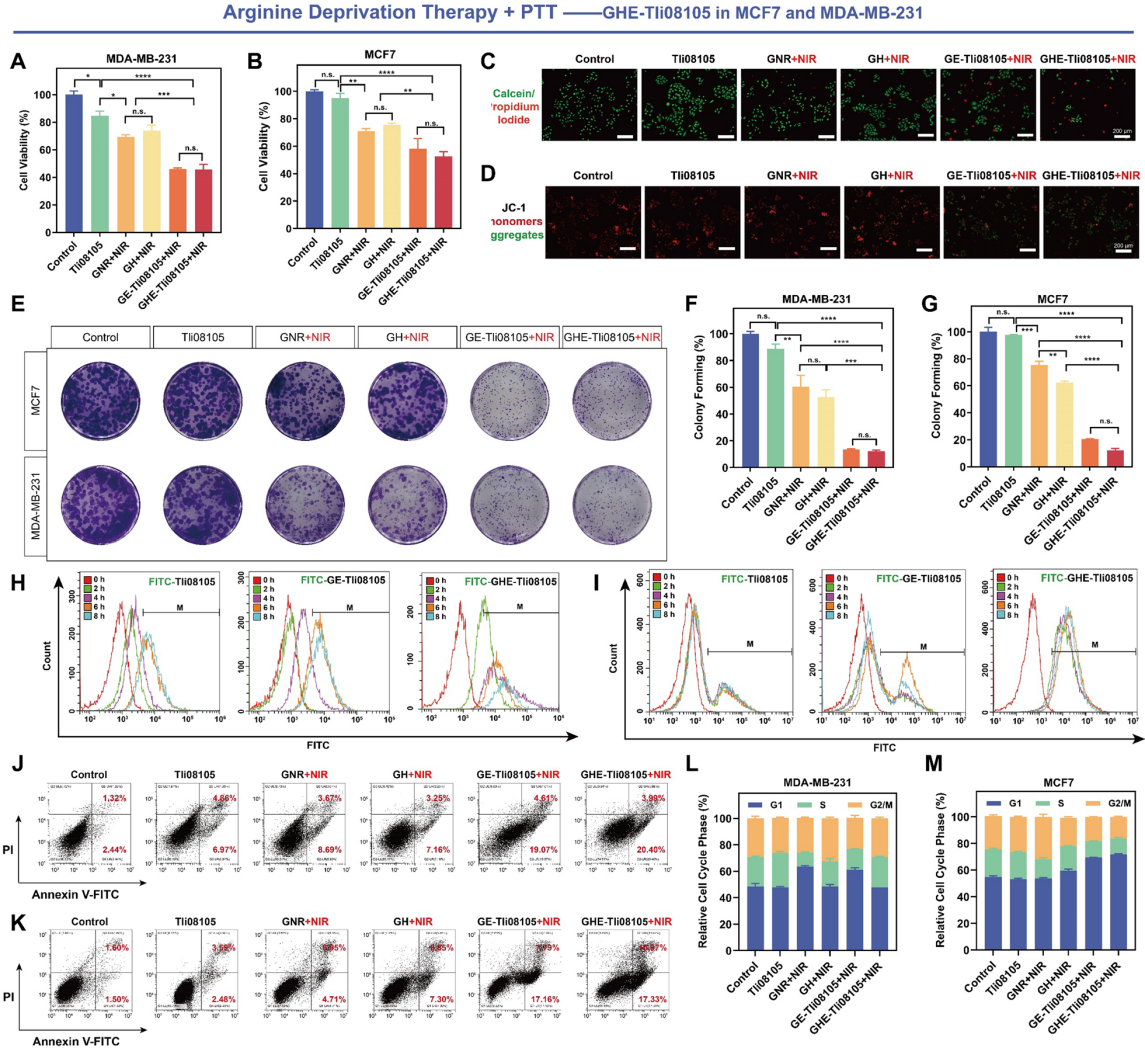
In vitro anticancer efficacy of arginine deprivation-photothermal-therapeutic performance of GHE-Tli08105. MTT assays of Tli08105, GNR + laser, GH + laser, GE-Tli08105 + laser and GHE-Tli08105 + laser groups in **A** MDA-MB-231 and **B** MCF7 cells. Tli08105 content was fixed at 25 μg/mL, and equal amounts of GNR/GH contain the same photothermal conversion efficiency compared with GE/GHE. Data are shown as the mean ± SD (n = 3). **C** Fluorescence images of viable (green) and dead (red) MCF7 cells staining with Calcein-AM/PI after treatment with different samples (scale bar: 200 μm). **D** Mitochondrial membrane potential of MCF7 cells analysis using JC-1mitochondrial membrane dye (scale bar: 200 μm). The colony formation of MCF7 cells and MDA-MB-231 cells was **E** captured and **F**, **G** quantified after photothermal activation of thermophilic arginase Tli08105 (*p < 0.05; **p < 0.01, ***p < 0.001, ****p < 0.0001). Data are shown as the mean ± SD (n = 3). Flow cytometric quantitative analyses of the endocytosis amounts of the nanocatalysts with different architectures in **H** MDA-MB-231 and **I** MCF7 cells for different incubation periods (the nanocatalysts were labeled with FITC). Flow cytometric quantitative analyses for the apoptosis of Annexin V-FITC/PI co-stained **J** MDA-MB-231 and **K** MCF7 cells after co-incubation with different samples. Quantitative flow cytometry analysis of the **L** MDA-MB-231 and **M** MCF7 cells stained with PI. Data are shown as the mean ± SD (n = 3)

**Fig. 6:**
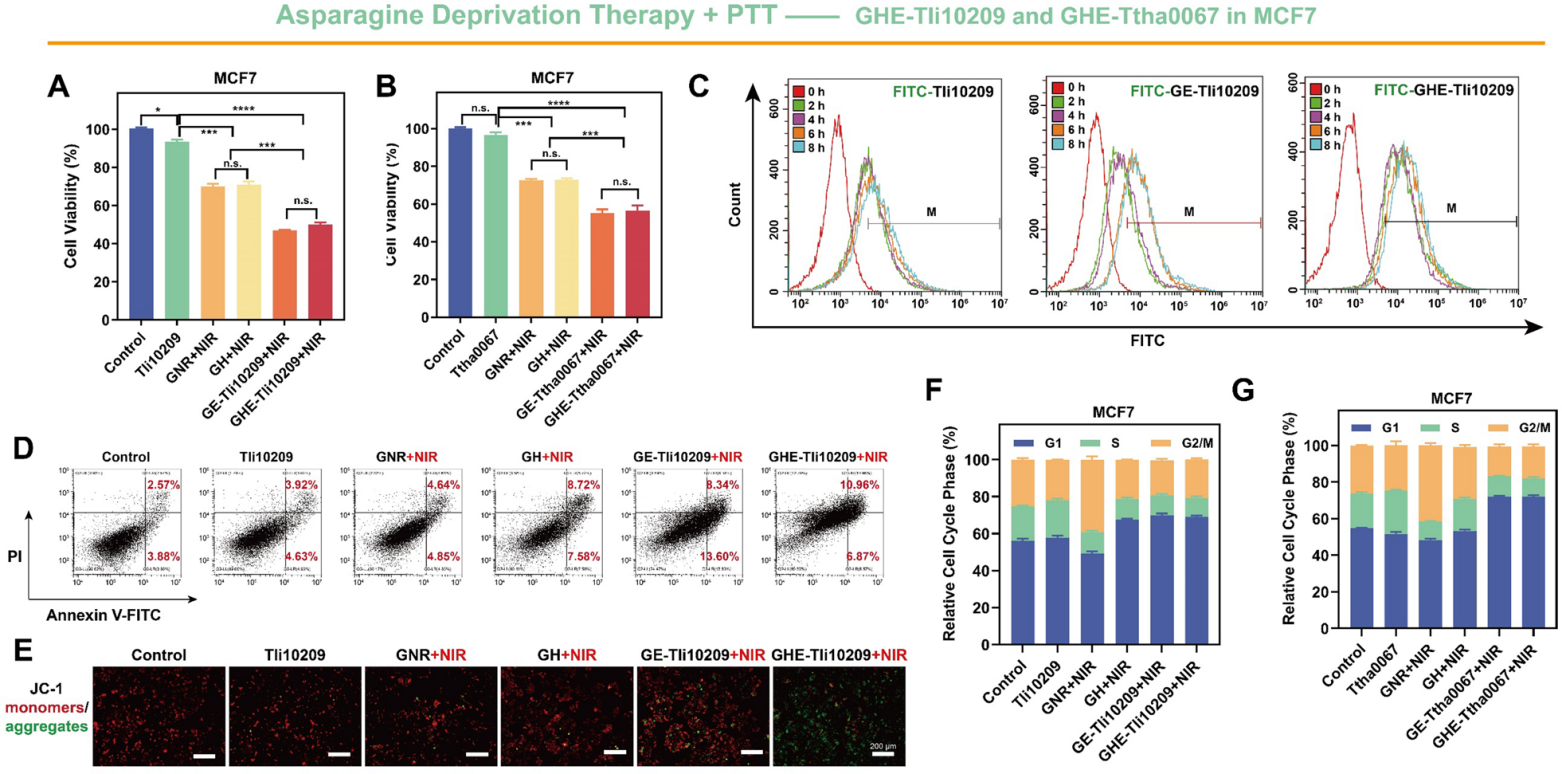
In vitro anticancer efficacy of asparagine deprivation-photothermal-therapeutic performance of GHE-Tli10209/Ttha0067. Induction of cell-proliferation effect in MCF7 cells through the MTT assay by GE/GHE nanocatalysts based on **A** Tli10209 and **B** Ttha0067. The thermozyme content was fixed at 25 μg/mL, and equal amounts of GNR/GH contain the same photothermal conversion efficiency compared with GE/GHE (*p < 0.05; **p < 0.01, ***p < 0.001, ****p < 0.0001). Data are shown as the mean ± SD (n = 3). **C** Flow cytometric quantitative analyses of the endocytosis amounts of the nanocatalysts with different architectures in MCF7 cells for different incubation periods (the nanocatalysts were labeled with FITC). **D** Flow cytometric quantitative analyses for the apoptosis of Annexin V-FITC/PI co-stained MCF7 cells after co-incubation with different nanocatalysts. **E** Fluorescence microscopy images of JC-1-labeled MCF7 cells treated with different samples. Scale bar: 200 μm. Cell cycle analysis by flow cytometry in MCF7 for the GHE-photothermal catalytic therapy with PI staining based on **F** Tli10209 and **G** Ttha0067. Data are shown as the mean ± SD (n = 3)

Many studies have shown that metabolic differences between cancer and normal cells determine the sensitivity of enzyme therapies, and cell death was also found to occur in both MCF7 and MCF10A cells after arginine deprivation. Therefore, clear cell identification needs to be incorporated to ensure targeted enzyme therapy [66, 71, 72]. To explore the cellular uptake of nanocatalysts, this study was visualized and quantified using GHE loaded with highly fluorescent FITC-Thermozyme (Figs. 5H, I, 6D and Additional file 1: Fig. S12). It was observed that GHE-Tli10209 was rapidly taken up into the cytoplasm, as evidenced by the strong green fluorescence within 0–8 h (Additional file 1: Fig. S12C). This observation was further supported by the quantitative analysis of nanoparticle cellular internalization (Fig. 6D). Comparing the presence/absence of HA further elucidated the targeting ability of GHE in MCF7 and MDA-MB-231 cells. The high density of negative charges on the surface of cancer cells made electrostatic adsorption of GE nanocatalysts and free thermozymes challenging [46]. HA, as a functional targeting agent for inert carriers, enhances cellular uptake by ligand binding to tumor cell surface markers [42]. Moreover, Xu et al. have shown that cancer cells exhibit stronger acidity and higher GSH and HAase favoring degradation of HA coatings on GNR [27], which may enhance toxicity by boosting substrate affinity.

The synergistic anticancer effect was next determined by MTT assay (Figs. 5A, B, 6A, B) to verify the cytostatic proliferative capacity of all components independently and/or in combination. This process ensured that the concentration of each component was equivalent to the final GHE formulation to assess the combinatorial effect of single elements. It was found that GHE nanocatalysts activated by NIR, were more cytotoxic to MCF7 and MDA-MB-231 cells than equivalent concentrations of free thermozymes. Compared to PTT alone (GNRs), photothermally triggered AAD showed stronger cell killing. In all three thermozyme-represented GHE models, it was clearly demonstrated that NIR remotely manipulated the mutual and synergistic anticancer effects of thermozymes with GNRs rather than single element induction.

Propidium iodide (PI) and Calcein-AM were used to stain dead and live cells, respectively, to facilitate the observation of anticancer effects (Additional file 1: Fig. S11). Red fluorescence was stronger in GE-/GHE-Tli08105 + NIR-treated cells than free Tli08105 in MCF7 cells, indicating a significant decrease in cell viability (Fig. 5C). Additionally, we observed that MCF7 cells changed from the typical cobblestone-like phenotype (strong intercellular attachment) to a more compact round-shape with reduced adhesion capacity after exposure to GHE + NIR. Under the microscope, most cancer cells were unable to adhere to the wall after the catalytic-photothermal interaction (Additional file 1: Fig. S11B). These findings were further supported by the results from the 7-day colony formation assays, which showed a significant reduction in the colony generation status induced by the tumor cell clusters growth (Figs. 5E–G), and Additional file 1: Fig. S13). This highlights the preferential selectivity and specific activation of our designed nanocatalysts, which may yield enhanced therapeutic advantages due to variations in the physiological-expression profiles of genes and proteins of cancer versus normal cells [73, 74]. Collectively, the above points demonstrate the anticancer benefits of GHE nanocatalyst.

### Induced apoptosis and cell cycle arrest by GHE in vitro

Intracellular AA deficiency results in nutritional starvation and endostatic imbalance, which inhibits protein synthesis and accelerates programmed tumor cell death [40]. Many studies have investigated the anticancer mechanism of AADT in various cellular and animal models, finding that it mainly induces cell cycle arrest and apoptosis [36, 75]. To identify the apoptotic population in GHE + NIR-treated breast cancer cells, we concentrated on the primary indicator of apoptosis, phosphatidylserine ectopia (Figs. 5J, K, 6D, Additional file 1: Figs. S14, S15). Compared to untreated cells, GHE-Tli08105 + NIR cultured MCF7 cells had a higher Annexin-V + count (28.3%), higher than GE-Tli08105 + NIR (24.9%) **(**Fig. 5K**)**. NIR-triggered Asn deprivation also showed considerable apoptosis compared with PTT alone **(**Fig. 6D**)**. This suggests that the catalytic-photothermal effect exacerbates apoptosis. Drug combinations are essential in anticancer therapy, as in the case of combined chemotherapeutic agents, which enhance cell death events [36].

In response to the stress of nutrient deprivation, mitochondria are essential in the elevated demand for energy and anabolism. We used a JC-1 fluorescent probe to monitor changes in the mitochondrial membrane potential (MMP). Normally growing breast cancer cells showed a high MMP (strong red fluorescence) (Additional file 1: Fig. S16). However, GHE-Tli08105 + NIR stimulated Arg-deprivation as well as GHE-Tli10209 + NIR triggered Asn-deprivation, significantly increasing the green fluorescence (Figs. 5D, 6E**)**. MMP dissipation triggered by focal hyperthermia was observed here and was associated with massive intracellular disruption of the membrane system, favouring apoptosis events [76, 77]. Additionally, the large amount of green fluorescence in an elliptical distribution around the nucleus implied that mitochondria were damaged. AA deficiency affects the bioenergetics and integrity of mitochondria [66]. Heat release and nutrient deficiency of the microenvironment may disrupt cells into a necrotic or apoptotic state.

The anti-proliferation mechanism of amino acid deficiency-induced tumor cells, cell cycle arrest, is considered to be closely associated with apoptotic and necrotic states [40, 65]. To analyze the effects of GHE + NIR-treatment on cell cycle arrest in MCF7 cells, we used flow cytometry with PI labelling to measure the distribution of DNA content in the total cell population (Additional file 1: Fig. S17). Our results showed that GE/GHE nanocatalysts + NIR-treated MCF7/MDA-MB-231 cells had significantly reduced numbers in the S and G2/M phases, and the combination induced cell cycle arrest in the G1 phase (Figs. 5L, M, 6F and G). Furthermore, photothermal stimulation of GNRs resulted in G2/M phase cell cycle arrest [78]. These findings suggest that nanocatalysts can enhance the effectiveness of treatment by inhibiting cell proliferation, promoting apoptosis, and causing cell cycle arrest.

### NIR-triggered AADT + PTT inhibited migration and invasion of surviving cells

**Fig. 7:**
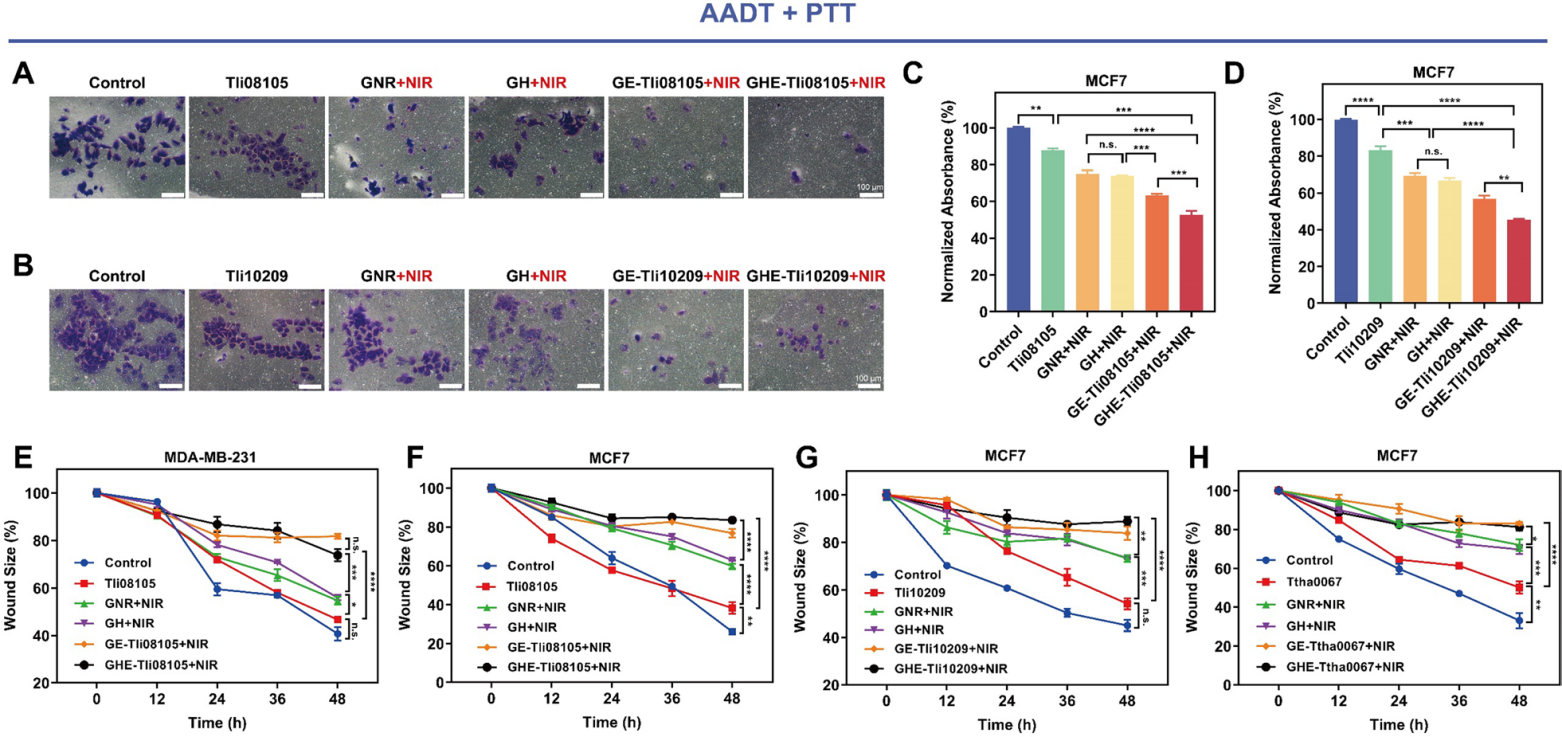
AADT + PTT triggered by NIR inhibit cell migration and invasion in vitro. **A**–**D** The effect of thermozyme and GE/GHE nanocatalysts on cell invasion as measured by transwell assays. Scale bar: 100 μm. **E**–**H** The effect of thermozyme and GE/GHE nanocatalysts on cell migration which was measured via wound healing assays within 48 h. Data are shown as the mean ± SD (n = 3). (*n.s.*, not significant; *p < 0.05; **p < 0.01, ***p < 0.001, ****p < 0.0001)

The primary obstacles to curing breast cancer are the invasion of regional lymphatic arteries and veins as well as distal organ metastases [79]. Exploring the catalytic-photothermal effect on the motility behaviour of tumor cells is thus extremely important in malignancy prevention. The migration ability after treatment was tested using a classical in vitro monolayer-cell wound healing model. Cell motility was unaffected by Tli08105 treatment alone. In contrast, Arg deprivation combined with photothermia only had a slow healing rate anchored to the central region (Fig. 7(E–F), and Additional file 1: Figs. S19(A–B) in MCF7 and MDA-MB-231 cells, similar to NIR-triggered Asn consumption (Fig. 7(G–H), and Additional file 1: Figs. S19(C–D) in MCF7 cells. Precise activation of thermozymes can participate in cellular activities efficiently and affect the viability, adhesion, and invasion of cancer cells, as well as the in-depth study of normothermic enzyme preparations [80]. Deprivation of Arg, the precursor of NO, significantly inhibited the migration of cancer cells. Furthermore, the consumption of AA as a contributor to local adhesion had a positive effect on damaging cell adhesion.

Tumor metastasis was also associated with detachment from the parent and invasion with extracellular matrix (ECM) adhesion. To validate this, we used the polycarbonate membrane of a Transwell chamber to simulate ECM. The catalytic-photothermal interaction mediated by GE/GHE nanocatalysts + NIR impaired the rate of cellular digestion of the matrix; the significant reduction in cell numbers implied that invasion capacity was inhibited (Fig. 7A–D, and Additional file 1: Fig. S18). Various intracellular AAs are of particular significance for collagen synthesis, cell proliferation, inflammation and wound healing. Rapid nutrient deficiency affects the endostatic restoration and the secretion of degradative enzymes, thereby affecting cell invasion [7, 81]. These findings suggest that AADT, triggered by local hyperthermia, may be an excellent candidate for regulating cell migration.

### Anti-tumor efficacy and biosafety evaluation in vivo

**Fig. 8:**
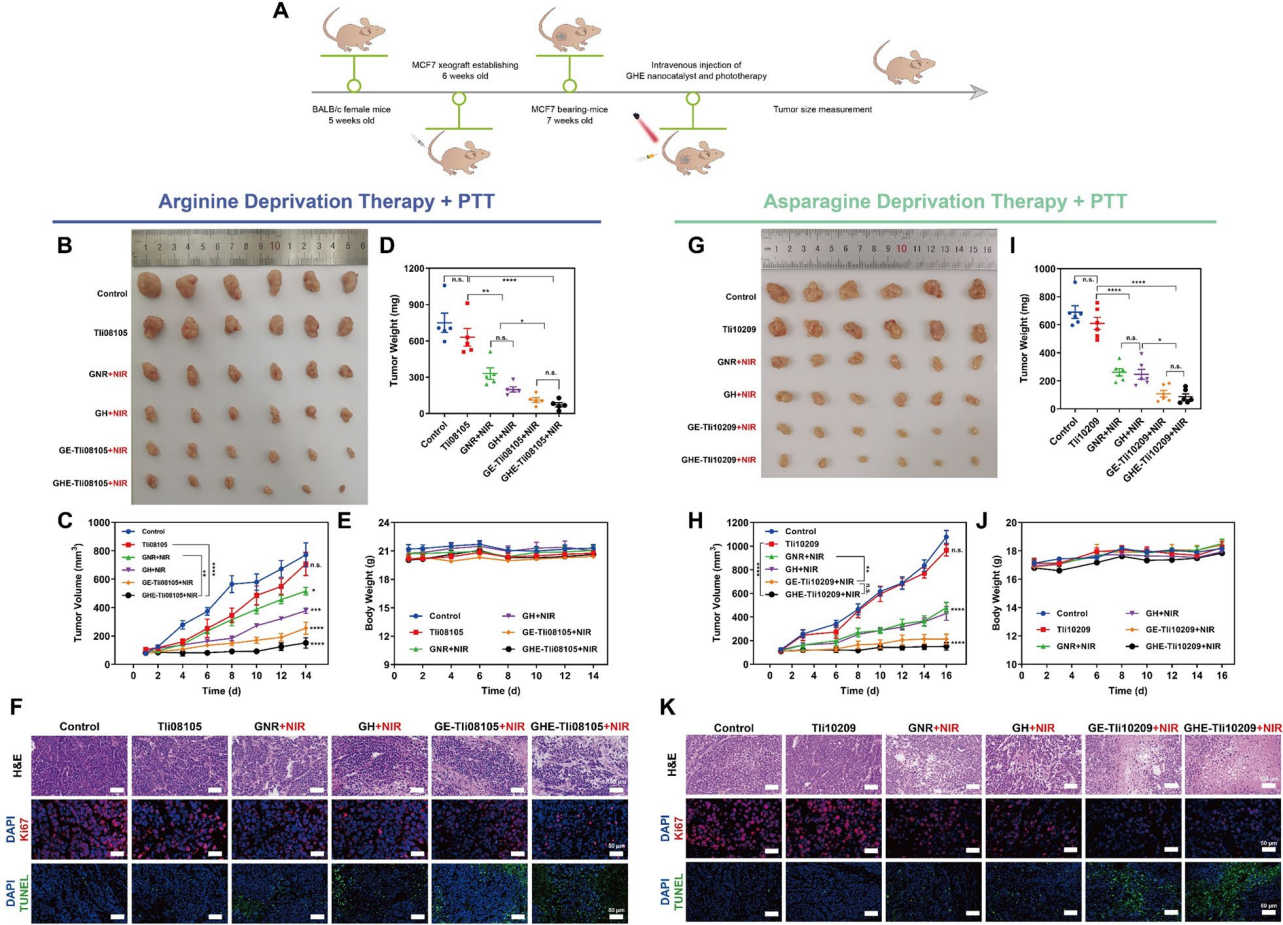
In vivo NIR-triggered photothermal-enhanced catalytic therapy of MCF7 xenograft tumor-bearing BALB/c nude mice. **A** Schematic illustration of the therapeutic protocol. **B**, **G** Digital photographs of the dissected tumors. The relative tumor volumes of MCF7 tumor-bearing mice treated with various **C** Tli08105 and **H** Tli10209-based treatments through *i.v.* modes. Tumor weights of each group after NIR-triggered locoregional **D** arginine and **I** asparagine deprivation-photothermal therapy. Body weight curves of nude mice during **E** Tli08105- and **J** Tli10209-photothermal synergistic therapy. **F, K** H&E, Ki67 and TUNEL staining of the tumor tissues harvested from corresponding mice after various treatments. The scale bars are (H&E) 100 μm, and (Ki67, TUNEL) 50 μm, respectively

GHE with excellent in vitro photothermal-catalytic effects may indicate potential in vivo applications. To explore this, NIR-triggered Arg- and Asn-deprivation modes were selected and tested in MCF7 xenograft BALB/c nude mice **(**Fig. 8A**)**. An injection frequency of every 3 days was chosen, consistent with clinical phase III (recombinant *E. coli* asparaginase) [2, 11], with no weight loss observed in the animals during the treatment (Fig. 8E, J). In mice treated with GHE-Tli08105 + NIR *i.v.* injection, tumors were significantly reduced in size (151.7 mm^3^) after treatment (Fig. 8C) compared to the low-activity free Tli08105 group, indicating the synergistic effect of Arg‒deprivation and photothermal transduction in vivo. Similarly, Asn‒deprivation by GHE-Tli10209 + NIR had an inhibition rate of 85.9% (Fig. 8H). PTT, a popular treatment modality, also showed an excellent tumor suppression efficiency (55.7%). Furthermore, it was observed that the targeted combination (GHE + NIR) reduced tumor mass compared to single treatment (Fig. 8D, I), demonstrating the therapeutic advantages of catalytic-photothermal synergy.

To examine the pathology of organs and tumors, histological images of dissected tissues were obtained. Haematoxylin and eosin (H&E) staining images of tumor slices revealed that GHE-Tli08105 + NIR-treated tumor cells were significantly damaged, with nuclear shrinkage and decreased density (Fig. 8F). The loose arrangement of tumor cells implied inhibited growth, as well as apoptosis and/or necrosis **(**Fig. 8K**)**. Bright green fluorescence was observed in the terminal deoxynucleotidyl transferase-mediated dUTP nick end labelling (TUNEL) image, indicating massive cell ablation. Additionally, immunofluorescence staining analysis of Ki67 (cell proliferation-associated) expression in transplanted tumor tissues showed a slower proliferation rate of tumors treated with GHE + NIR, indicating low in situ differentiation.

**Fig. 9:**
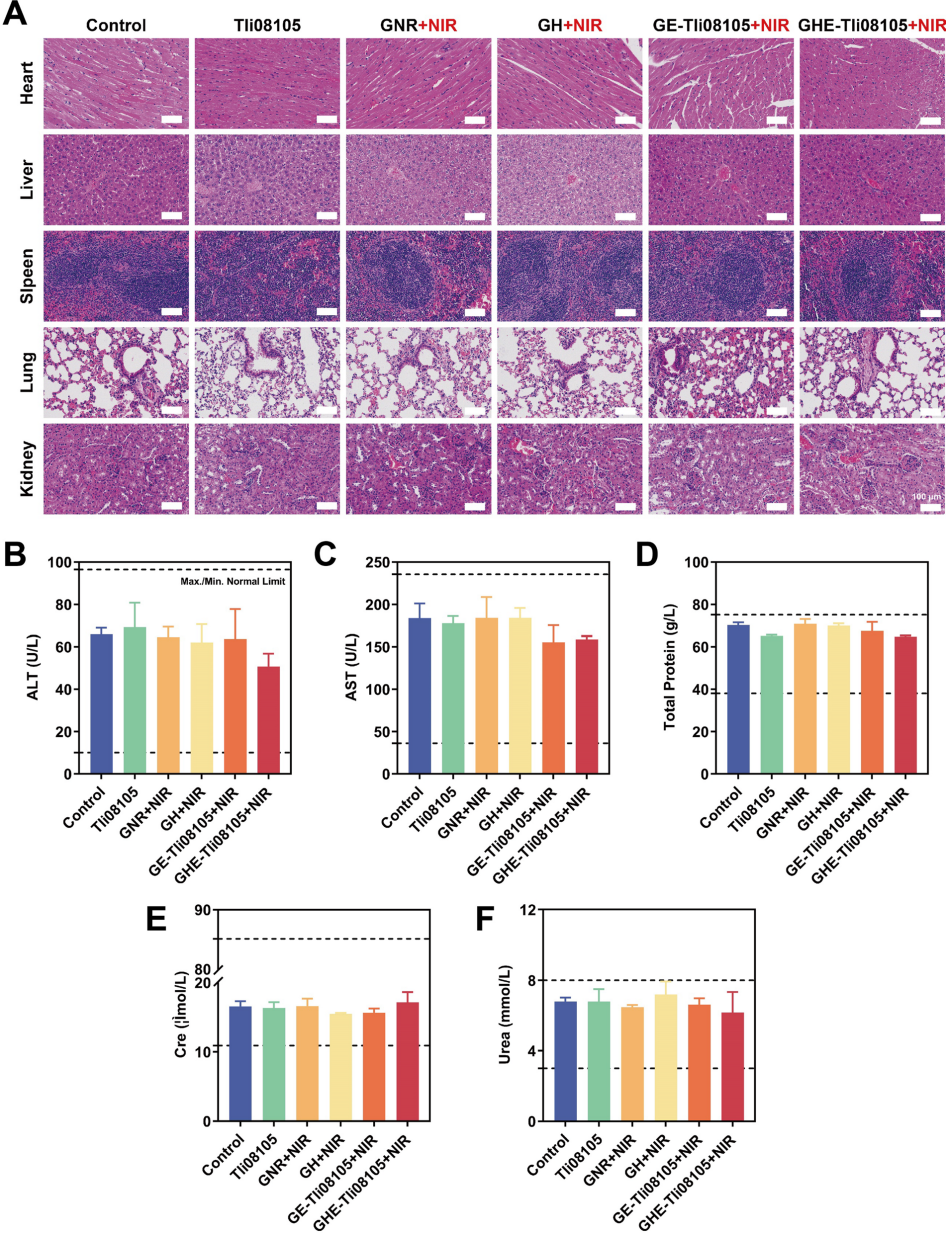
In vivo biosafety evaluation of GHE nanocatalysts. **A** H&E staining images of the vital organs (heart, liver, spleen, lung and kidney) of nude mice in different treatment groups. Scale bar: 100 μm. **B**–**F** Blood biochemical profile tests after synergistic Tli08105-catalytic-PTT, including alanine aminotransferase (ALT), aspartate aminotransferase (AST), total protein, creatinine (Cre), and urea

To assess biosafety, haematological indices of the tumor-bearing mice were evaluated, including ALT and AST (related to liver function), urea and creatinine (related to renal function), and total protein levels, which were within normal limits (Fig. 9B–F and Additional file 1: Fig. S21). Moreover, H&E images showed no histomorphological damage with organic lesions in the main organs (heart, liver, spleen, lungs and kidneys) of dissected mice (Fig. 9A, and Additional file 1: Fig. S20). Damage to surrounding healthy tissue was minimized by precisely irradiating the localized site with an external laser that can be operated in real time. In addition, the temperature of photothermal conversion can reach 42–43 ℃ at the tumor site. Cancer cells were more susceptible to other therapeutic modalities, with minimal impact on normal cells [82]. Conjugated thermozymes are affected by the activation of the internal and thermal energy of the transduction, which can better support the transformation of favorable catalytic conformations [23, 44]. These findings confirmed that the rapid and sustained induction of AA deprivation by GHE + NIR is biosafe, thermostable and remotely light manipulable, and has the potential to be used for the treatment of other nutrient deficient solid tumors.

## Conclusion

In summary, we have constructed a spatiotemporally enhancible GHE nanocatalyst for NIR-triggered concomitant PTT, enabling precise cellular amino acids deprivation with long-term tumor suppression. This novel strategy is based on the catalytic ability of remotely activated thermozymes, which are specifically induced only in cancer cells, providing a basis for precise, biostable and controlled enzyme therapy. The general applicability of GHE nanocatalysts utilizing three unique screened thermophilic AA hydrolases from thermophilic bacteria was confirmed to demonstrate significant efficacy both in vitro and in vivo*.* The advantages of NIR-enhanced thermocatalytic-photothermal therapy over conventional enzyme therapies are as follows: (1) Thermozymes exhibit temperature-limited lower catalytic activity during in vivo circulation, thus preventing off-target toxicity; (2) HA promotes precise cancer cell endocytosis; (3) Photothermal and internal energy transfer enhances thermal-dependent catalysis, allowing for rapid and sustained AADT to efficiently inhibit the proliferation and migration of cancer cells; (4) PTT synergistically facilitates the sensitivity of cancer cells to other treatments and accelerates cell death in multipath. This demand-oriented, remotely excitable and long-acting nanocatalyst has the potential to provide new ideas for the design of multifunctional high-efficient theranostic enzyme drugs.

### Supplementary Information

**Additional file1: **There are experimental materials and methods, 18 figures and 1 table in total, which are as important as the figures in main article as the supplementary content of this manuscript. **Table S1.** Primers sequence for arginase and asparaginases. **Figure S1.** Enzymatic properties of thermozymes. **Figure S2.** Thermophilic enzymatic reaction time course curve. **Figure S3. **Michaelis-Menten kinetics analysis of thermozymes. **Figure S4.** FTIR spectra of HA, HA-Cys and GNR-HA. **Figure S5.** SEM images of GH, GE-Tli08105, GE-Tli10209, GE-Ttha0067, GHE-Tli10209, GHE-Ttha0067. **Figure S6.** UV-Vis absorption spectra of Ttha0067 before and after assembly with GNR or GH. **Figure S7.** The biostability of GE/GHE nanocatalysts. **Figure S8.** The hydrodynamic size of nanocatalyst in different medium. **Figure S9. **The storage stability of thermozymes and correlative GE/GHE nanocatalysts.** Figure S10.** Relative viability of MCF10A cells incubated with different concentration of thermozyme/nanocatalyst. **Figure S11.** Fluorescence images of viable and dead breast cancer cells (MDA-MB-231 and MCF7 cells) treated with different samples. **Figure S12.** *In vitro* cellular uptake assessment. **Figure S13.** Colony formation of MCF7 cells effected by thermophilic asparaginases and NIR-triggered nanocatalysts. **Figure S14.** Flow cytometric quantitative analyses of Annexin V-FITC/PI co-stained MDA-MB-231 and MCF7 cells after co-incubation with different samples. **Figure S15.** Apoptosis analyses of MCF7 cells cultured with GHE-Ttha0067 + NIR. **Figure S16.** Mitochondrial membrane potential of MDA-MB-231 and MCF7 cells analysis using JC-1 mitochondrial membrane dye. **Figure S17.** Cell cycle analysis by flow cytometry for the GHE-photothermal catalytic therapy with PI staining. **Figure S18.** Invasion ability of MDA-MB-231 and MCF7 cells after different treatments. **Figure S19.** Representative images of cell migration of MDA-MB-231 and MCF7 cells after different treatments *via* wound healing assays. **Figure S20.** H&E staining images of major organs. **Figure S21.** Blood biochemical profiles after synergistic Tli10209-catalytic-PTT.

## Data Availability

The datasets and analysed during the current study are available from the corresponding author on reasonable request.

## Abbreviations

AADT: Amino acid deprivation therapy
ALT: Alanine aminotransferase
ASNS: Asparagine synthase
AST: Aspartate aminotransferase
Cre: Creatinine
CTAB: Hexadecyltrimethylazanium bromide
DMEM: Dulbecco’s modified eagle’s medium
EDC: 1-(3-Dimethylaminopropyl)-3-ethylcarbodiimide hydrochloride
*E.coli*: *Escherichia coli*
FBS: Fetal Bovine Serum
FTIR: Fourier Transform Infrared Spectra
GNR: Gold nanorod
HA: Hyaluronic acid
GHE: GNRs@HA@Thermozyme
ISR: Integrated stress response
MMP: Mitochondrial membrane potential
MTT: 3-(4,5-Dimethylthiazol-2-yl)-2,5-diphenyltetrazolium bromide
NHS: N-Hydroxysuccinimide
NIR: Near-infrared
PTT: Photothermal therapy
RES: Reticuloendothelial system
SEM: Scanning electron microscopy
TEM: Transmission electron microscopy
TUNEL: Terminal deoxynucleotidyl transferase-mediated dUTP nick end labelling
TP: Total protein
