# Supplementary material for: Selective enhanced cytotoxicity of amino acid deprivation for cancer therapy using thermozyme functionalized nanocatalyst
Source: J Nanobiotechnology. 2024 Feb 7;22:53. doi: 10.1186/s12951-024-02326-6 (PMC10848425; doi:10.1186/s12951-024-02326-6)
Supplement: Supplementary file 1 — Additional file1: There are experimental materials and methods, 18 figures and 1 table in total, which are as important as the figures in main article as the supplementary content of this manuscript. Table S1. Primers sequence for arginase and asparaginases. Figure S1. Enzymatic properties of thermozymes. Figure S2. Thermophilic enzymatic reaction time course curve. Figure S3. Michaelis-Menten kinetics analysis of thermozymes. Figure S4. FTIR spectra of HA, HA-Cys and GNR-HA. Figure S5. SEM images of GH, GE-Tli08105, GE-Tli10209, GE-Ttha0067, GHE-Tli10209, GHE-Ttha0067. Figure S6. UV-Vis absorption spectra of Ttha0067 before and after assembly with GNR or GH. Figure S7. The biostability of GE/GHE nanocatalysts. Figure S8. The hydrodynamic size of nanocatalyst in different medium. Figure S9. The storage stability of thermozymes and correlative GE/GHE nanocatalysts. Figure S10. Relative viability of MCF10A cells incubated with different concentration of thermozyme/nanocatalyst. Figure S11. Fluorescence images of viable and dead breast cancer cells (MDA-MB-231 and MCF7 cells) treated with different samples. Figure S12. In vitro cellular uptake assessment. Figure S13. Colony formation of MCF7 cells effected by thermophilic asparaginases and NIR-triggered nanocatalysts. Figure S14. Flow cytometric quantitative analyses of Annexin V-FITC/PI co-stained MDA-MB-231 and MCF7 cells after co-incubation with different samples. Figure S15. Apoptosis analyses of MCF7 cells cultured with GHE-Ttha0067 + NIR. Figure S16. Mitochondrial membrane potential of MDA-MB-231 and MCF7 cells analysis using JC-1 mitochondrial membrane dye. Figure S17. Cell cycle analysis by flow cytometry for the GHE-photothermal catalytic therapy with PI staining. Figure S18. Invasion ability of MDA-MB-231 and MCF7 cells after different treatments. Figure S19. Representative images of cell migration of MDA-MB-231 and MCF7 cells after different treatments via wound healing assays. Figure S20. H&E staining image [file 12951_2024_2326_MOESM1_ESM.docx]

**Additional file Information**

**Additional file 1**

**Selective enhanced cytotoxicity of amino acid deprivation for cancer therapy using thermozyme functionalized nanocatalyst**

Xiuhui Tang ^a^, Lijuan Zhang ^b^, Mingwang Huang ^a^, Fang Wang ^a^, Guiqiu Xie ^b^, Rui Huo ^b^, Renjun Gao ^a,*^

*^a^ Key Laboratory for Molecular Enzymology and Engineering of Ministry of Education, School of Life Sciences, Jilin University, Changchun 130012, China*

*^b^* *School of Pharmaceutical Sciences, Jilin University, Changchun 130021, China*

*Corresponding author.

Tel.: +8618604313058; Fax: +8643185155200.

E-mail: [gaorj@jlu.edu.cn](mailto:quanshun@jlu.edu.cn) (R. Gao).

**Additional file Experimental**

1. **Cell lines and cell culture**

The breast cancer cell lines MCF7, MDA-MB-231 and the mammary epithelial cell line MCF10A were obtained from the American Type Culture Collection (ATCC; MD, USA). All cell lines underwent routine mycoplasma testing before being cultivated at 37 °C in a humidified incubator containing 5% CO_2_. Briefly, MCF7, MDA-MB-231 and MCF10A cells were grown in Dulbecco’s modified Eagle’s medium (DMEM; Gibco) with 10% fetal bovine serum (FBS; Kangyuan Co.).

1. **Bacterial Strains and Plasmids**

The genomic DNA of *Thermococcus litoralis* (JCM 8560) and *Thermus thermophilus* HB8 (JCM 10941) from the Japan Collection of Microorganisms (JCM) were used as PCR templates. For DNA manipulation and amplification, *Escherichia coli* (*E. coli*) DH5α strain was employed, while *E. coli* strain BL21 (DE3) was used to express asparaginases and arginase. The plasmid pET-28a (+) vector was purchased from Takara Biotechnology Co., Ltd. (Dalian, China) for DNA cloning and expression.

1. **Materials and reagents**

Materials required for the synthesis of GNRs and the reagents for the assay of arginase and asparaginase activities were purchased from Sinopharm Group Chemical Reagent Co., Ltd. (Beijing, China). All restriction enzymes, PrimeSTAR® HS (Premix), prestained protein ladder and DNA marker, and T4 DNA ligase were purchased from Takara Biotechnology Co., Ltd. (Dalian, China). The HighPure Plasmid miniprep kit, Universal DNA purification kit, BCA protein quantification kit, and kanamycin monosulfate were supplied by Bioteke (Beijing, China). The Ni^2+^-NTA agarose resin column used for protein purification was provided by GE Healthcare (Uppsala, Sweden). DMEM was purchased from Gibco (Grand Island, NY). FBS was obtained from Kangyuan Co. (Beijing, China). The Calcein/PI Cell Viability/Cytotoxicity assay kit, the enhanced mitochondrial membrane potential assay kit with JC-1, the Colorimetric TUNEL Apoptosis Assay Kit, 4',6-diamidino-2-phenylindole (DAPI), 4% paraformaldehyde, Triton X-100, haematoxylin and eosin were purchased from Beyotime (Shanghai, China). The Annexin V-PI Apoptosis Detection Kit and Cell Cycle analysis kit were obtained from BestBio Biotechnology (Nanjing, China). Fluorescein isothiocyanate (FITC) and trypsin were acquired from Sigma‒Aldrich. 3-(4,5-Dimethyl-2-thiazolyl)-2,5-diphenyl tetrazolium bromide (MTT) was obtained from Amersham (Solon, OH). A Ki67 protein immunohistochemistry Kit was obtained from Cell Signaling Technology. The DNA amplification primers used for the construction of thermozymes were synthesized by Comate Bioscience Co., Ltd. Other reagents not otherwise specified were purchased from Yuanye Biotechnology Co., Ltd. (Shanghai, China).

1. **Cloning, expression, and purification of arginase and asparaginases**

Briefly, the corresponding primers shown in Table S1 were constructed in accordance with the DNA sequences reported by GenBank, and PrimeSTAR® HS (Premix) was used as the polymerase for DNA amplification of the genes *OCC_08105*, *OCC_10209*, and *TTHA0067* (837 bp, 1038 bp, and 981 bp, respectively). The purified amplified DNA fragments were ligated with the pET-28a (+) vector after being cut with the corresponding restriction endonucleases and then transformed into *E. coli* DH5α competent cells. The obtained recombinant plasmids were next transformed into *E. coli* BL21 (DE3) cells after being validated by DNA sequencing. The expansion of recombinant cells was carried out at 37 °C in liquid Luria-Bertani (LB) medium containing 10 mg/mL kanamycin. Protein expression was induced by the addition of 0.3 mM Isopropyl-beta-D-thiogalactopyranoside (IPTG) when its optical density (OD) at 600 nm was 1.0 and incubated at 20 °C in a ZHWY-2102C constant temperature incubation shaker (Shanghai Zhicheng Analytical Instrument Manufacturing Co., Ltd.) at 120 rpm for 12 h. Cryogenic centrifugation was used to harvest the induced cells after one wash in 50 mM phosphate buffer (pH 7.0). According to the Ni^2+^-NTA agarose resin column specification, protein purification was performed using the imidazole gradient elution method. The protein molecular weights of the three thermophilic enzymes were detected by 12% sodium dodecyl sulfate‒polyacrylamide gel electrophoresis (SDS‒PAGE), and the protein concentrations were quantified via the Bradford method.

1. **Enzyme catalysis assay**

Arginase activity was quantified using the urea colorimetric assay (hydrolysate of L-arginine). The red-complex formed by urea, o-phthalaldehyde (oPA) and naphthylethylenediamine hydrochloride (NED) is proportional to the urea content. The hydrolysis reaction was carried out by adding 100 μL of 0.2 M L-arginine in 250 μL of 50 mM phosphate buffer for 2 min after the addition of arginase to the HH.W21 thermostatic water bath (Beijing Zhongxingweiye Instruments Co., Ltd., China). The reaction was subsequently terminated by adding 1.5 mL of prechilled chromogenic solution (2.4 M sulfuric acid, 200 mM boric acid, 6.4 mM *o*PA, 3.2 mM NED). The mixtures were incubated at 37 °C for 30 min for colouration, and the absorbance value was detected at 520 nm with an ultraviolet spectrophotometer UV-2700 (Shimadzu, Japan). One unit of arginase activity was defined as the amount of arginase used to hydrolyse 1 μmol L-arginine per minute.

Asparaginase activity was determined utilizing colorimetry of the reddish-brown complex formed by the interaction of NH_3_ under the hydrolysis of L-asparagine with the Nessler reagent (configuration of Nessler reagent: 7.0776 g potassium mercuric iodide and 14.0275 g potassium hydroxide were dissolved in Milli-Q water; and, after standing and cooling, the volume was fixed to 100 mL and stored at room temperature away from light, which is similar to the above concept. After preheating 200 μL of 50 mM phosphate buffer containing 100 μL of the substrate L-Asn (80 mM) in a water bath, asparaginase was added, and the reaction was hydrolysed for 5 min and then terminated with 100 µl of 1.5 M trichloroacetic acid (TCA). The chromogenic traction was carried out for 10 min at room temperature after the addition of 200 μL of supernatant and 1.4 mL of Milli-Q water that had been centrifuged to eliminate contaminants. To quantify NH_3_, the absorbance value at 450 nm was recorded. One micromolar of L-asparagine hydrolysed per minute was used as one unit of asparaginase.

1. **Cytotoxicity assay**

MCF10A cells were seeded in a 96-well plate at a density of 5.0×10^3^ cells/well, incubated for 24 h and subsequently treated with different concentrations of thermozyme/nanocatalyst (0-100 μg/mL) for 24 h. Treatment with MTT solution and determination of cell viability were performed as described above.

1. **Cellular uptake**

First, the 10 mL PBS solution containing 10 mg of thermozyme and 1 mg of fluorescein isothiocyanate (FITC) was coupled and stirred for 24 h at 4 °C in the dark. To remove unreacted chemicals, the mixture was dialyzed (MWCO: 7,000 Da) in ultrapure water overnight at 4 °C until the absorbance of the dialysate at 480 nm was 0. Finally, freeze-drying was used to obtain the lyophilized FITC-enzyme powder. Then, FITC-labeled thermozyme was used to synthesize binary or ternary nanocatalysts. Trypsin digestion of MCF7 or MDA-MB-231 cells was followed by an overnight culture in 12-well plates. Cells were treated with various FITC-labeled nanocatalysts for different lengths of time. After the entrance time had passed, the supernatant was discarded and the cells were washed twice with PBS before being observed under an IX73P1F fluorescence microscope (Olympus, Japan). The fluorescence intensity of FITC was quantified by flow cytometry (CytoFLEX, Beckman Coulter, USA) on trypsin-digested cells.

1. **Colony formation assay**

Similar to the above, prefabricated nanocatalysts were applied to treat MCF7 or MDA-MB-231 cells, which were cultivated in 12-well plates at a density of 10 × 10^4^/mL. To activate the thermozyme, cells underwent 2.0 W/cm^2^ NIR laser irradiation every 2 days for an additional 7 days of culture. All medium was removed, and the cells were rinsed 3 times with PBS. After the cells were fixed with 70% precooled ethanol at -20 °C for 1 h, the supernatant was removed and washed with PBS. Crystal violet dye, diluted to 0.2%, was used to stain cell colonies. The morphology of colony formation was photographed and recorded. Subsequently, the cell colonies were lysed with 33% glacial acetic acid, and the absorbance at 570 nm was recorded after centrifugation to remove impurities to quantify the colonies. The colony-forming ability of each group of cells was calculated based on the OD570. Colony formation efficiency was calculated as follows:

Colony formation efficiency (%) = Experimental group (OD570) / Negative control (OD570) × 100%

1. **Live/Dead Cell Staining**

Similar treatment was given to MCF7 or MDA-MB-231 cells as in the cytotoxicity assay. The Calcein/PI Viability/Cytotoxicity Assay Kit was used to determine the live/dead cell ratio immediately following the completion of NIR-activated deprivation of amino acids. This procedure was performed as instructed and captured under a fluorescence microscope.

1. **Mitochondrial membrane potential detection**

Simply put, the NIR-triggered treatment of MCF7 or MDA-MB-231 cells was comparable to that described. The fluorescent probe JC-1 was employed to detect the potential of mitochondria in cells. Fluorescence microscopy of the fluorescence signal of JC-1 as an aggregate/monomer in the mitochondrial matrix was used to determine the intensity of the mitochondrial membrane potential.

1. **Effect of cell migration and invasion ability**

MCF7 or MDA-MB-231 cells were precultured overnight in 12-well plates at a density of 20 × 10^4^/mL and treated to investigate on wound healing. When the confluence rate reached 90%, the cells were scratched with a 200 μL pipette tip. The wound healing width was observed and measured by microscopy at various times.

Identical treatment and NIR irradiation were executed in cell invasion assays. Cells were trypsinized and counted after the 24-hour treatment period. A total of 10,000 cells from different groups were dissolved in 200 µL DMEM containing 1% BSA and injected into the upper layer of the Transwell chamber, which was positioned on a 24-well plate. The lower layer of the Transwell chamber of the 24-well plate received an injection of 650 μL of DMEM complete medium containing 10% FBS. For an additional 24 h, the 24-well plate was incubated in a 37 °C, 5% CO_2_ atmosphere. After carefully removing the upper layer of cells from the Transwell chamber with sterile cotton swabs, the lower layer of cells was infiltrated with 70% precooled ethanol and preserved at -20 °C for 30 min. Likewise, the cells were stained with 0.2% crystal violet solution and captured microscopically. Quantitation was also taken by dissolving in 33% glacial acetic acid. The invasion of tumor cells was determined by comparison with the negative control group.

**Supplementary Table**

**Table S1. Primers sequence for arginase and asparaginases**

| **Protein** | **Enzyme** | **Resources** | **Gene** | **Gen-Bank** | **Primers (5’-3’)** |
| --- | --- | --- | --- | --- | --- |
| Tli08105 | arginase | *Thermococcus litoralis* (JCM8560) | *OCC_08105* | EHR79439.1 | sense: TGAGCCCATATGCTTTTTGGAATC  antisense: GGCTGGGAATTCTCATTTGGTTTT |
| Tli10209 | asparaginase | *Thermococcus litoralis* (JCM8560) | *OCC_10209* | EHR79842.2 | sense: ATCCGGCATATGAGCGAGAAAAGAA  antisense: TTGGCGGGATCCTTAACCCTCTATTT |
| Ttha0067 | asparaginase | *Thermus thermophilus* HB8 (JCM10941) | *TTHA0067* | BAD69890.1 | sense: TTAATACATATGAGCGCCGAGGTCCT  antisense: CTTACCGAATTCTCAGACCCAGACCA |

**Additional file Figures**


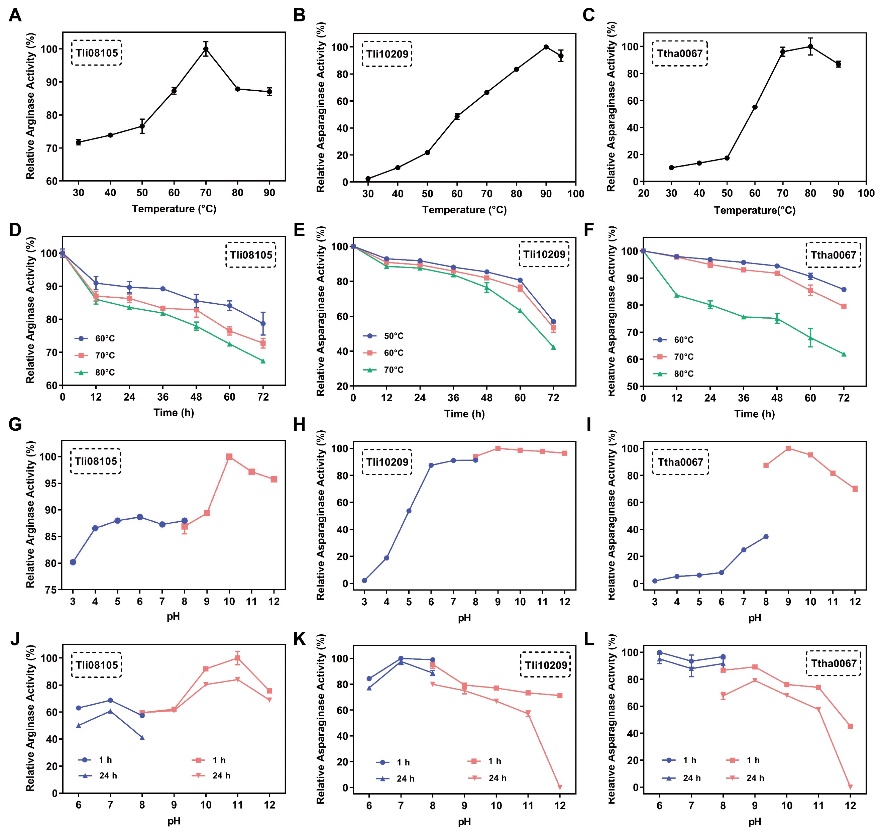


**Figure S1. Enzymatic properties of thermozymes.** Effect of temperature on **(A)** arginase Tli08105, **(B)** asparaginase Tli10209, and **(C)** asparaginase Ttha0067 activity in 50 mM phosphate buffer (pH 7.0). Thermostability of **(D)** Tli08105, **(E)** Tli10209 and **(F)** Ttha0067. The thermophilic arginase or asparaginase with a final concentration of 0.1 mg/mL was placed in a water bath at different temperatures for phase time. Samples were taken at the specified time and the residual enzyme activity was detected at the optimum temperature in 50 mM phosphate buffer (pH 7.5). Effect of pH on **(G)** Tli08105, **(H)** Tli10209, and **(I)** Ttha0067 activity. The determination conditions were as follows: for the hydrolysis of arginine or asparagine, at 37 °C; 50 mM acetic acid-sodium acetate buffer (pH: 3.0-8.0) or sodium bicarbonate-sodium hydroxide buffer (pH: 8.0-12.0). pH stability of **(J)** Tli08105, **(K)** Tli10209 and **(L)** Ttha0067. Residual activity was measured under different conditions by standard assays: in 50 mM acetic acid-sodium acetate buffer (pH: 6.0-8.0) after incubation for 1 h or 24 h at 25 °C, and in sodium bicarbonate-sodium hydroxide buffer (pH: 8.0-12.0) after incubation for 1 h and 24 h at 25 °C, respectively. Data are shown as the mean ± SD (n = 3).


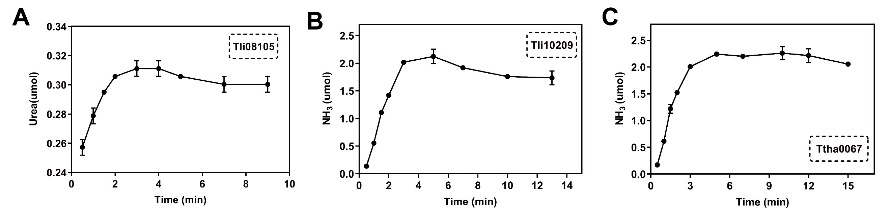


**Figure S2. Thermophilic enzymatic reaction time course curve.** Determined under optimal reaction conditions for thermophilic **(A)** Tli08105, **(B)** Tli10209, and **(C)** Ttha0067. Data are shown as the mean ± SD (n = 3).


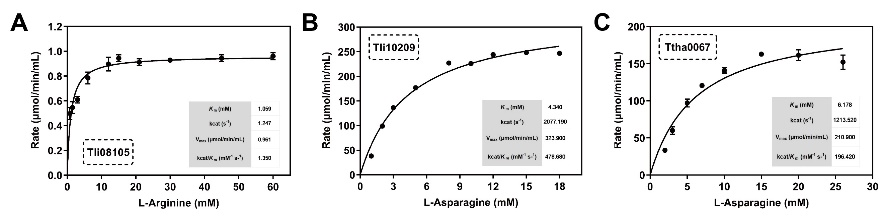


**Figure S3.** Michaelis-Menten kinetics analysis of the catalytic reactions by different thermozymes.


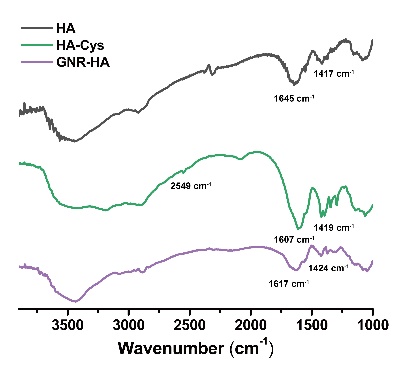


**Figure S4.** FTIR spectra of HA, HA-Cys and GNR-HA.


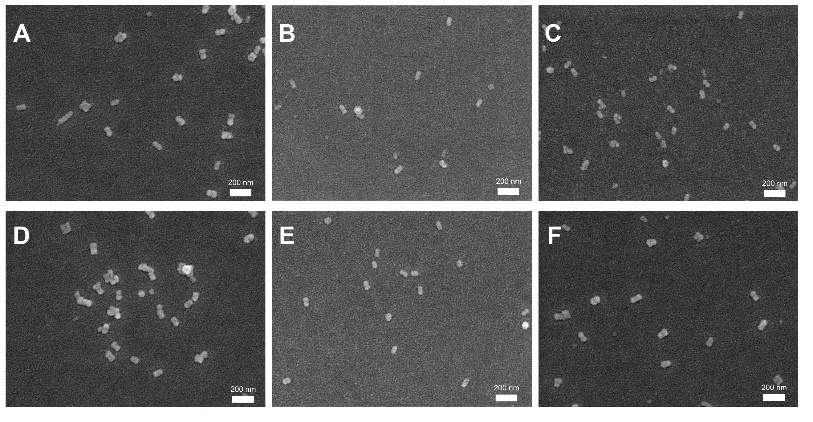


**Figure S5.** SEM image of **(A)** GE-Tli08105; **(B)** GE-Tli10209; **(C)** GE-Ttha0067; **(D)** GH; **(E)** GHE-Tli10209 and **(F)** GHE-Ttha0067. Scale bar: 200 nm.


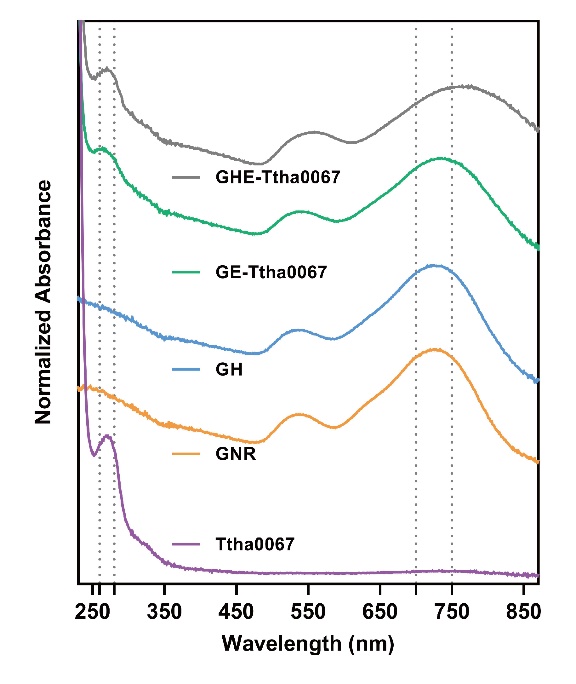


**Figure S6.** UV-Vis absorption spectra of Ttha0067 before and after assembly with GNR or GH.


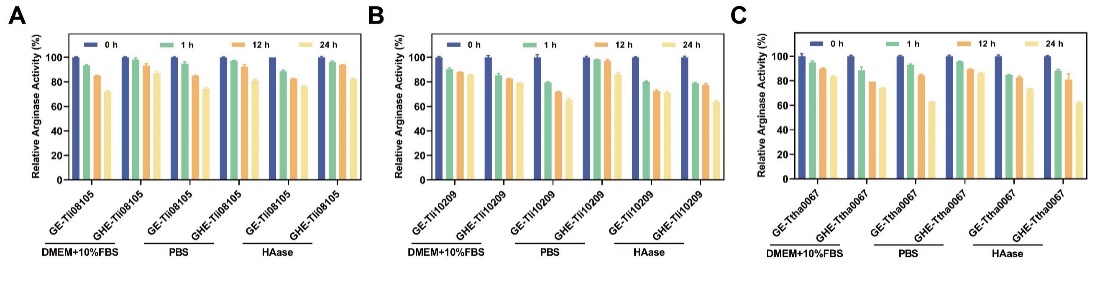


**Figure S7.** Based on **(A)** Tli08105, **(B)** Tli10209 and **(C)** Ttha0067, the biostability of GE/GHE nanocatalysts were measured by residual viability dissolved in DMEM (containing 10% FBS), PBS or HAase buffer with standing time. Data are shown as the mean ± SD (n = 3).

**
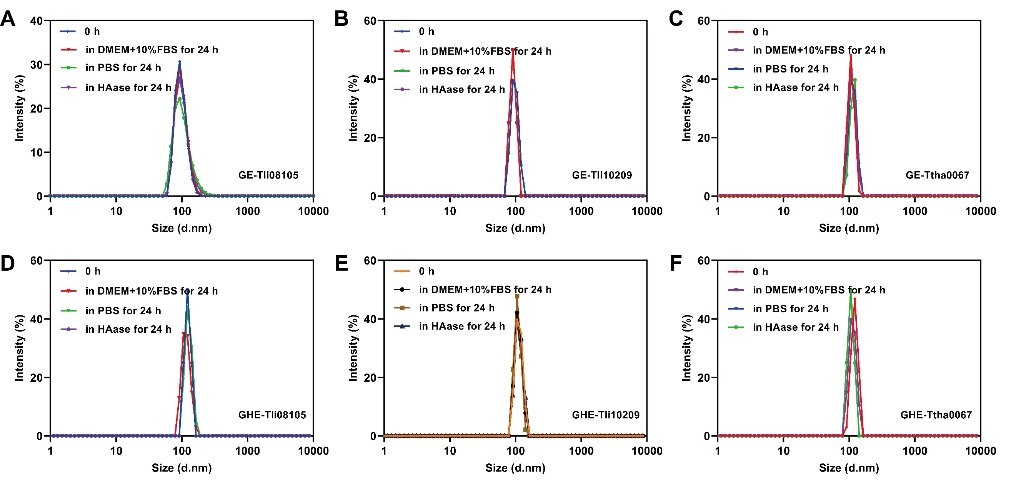
**

**Figure S8.** The hydrodynamic size of the nanocatalysts in DMEM (containing 10% FBS), PBS or HAase for 24 h.


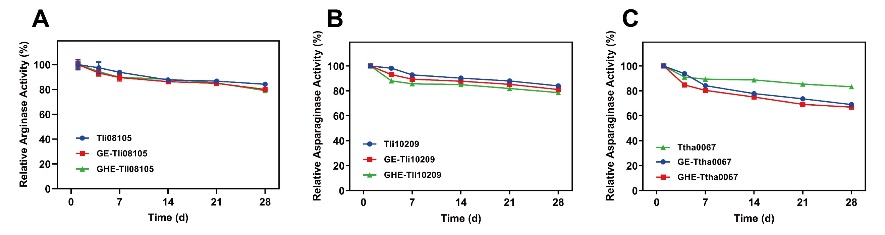


**Figure S9.** Storage stability of thermozymes and correlative GE/GHE nanocatalysts were determined for residual activity by periodic sampling stored at 4 °C in PBS.


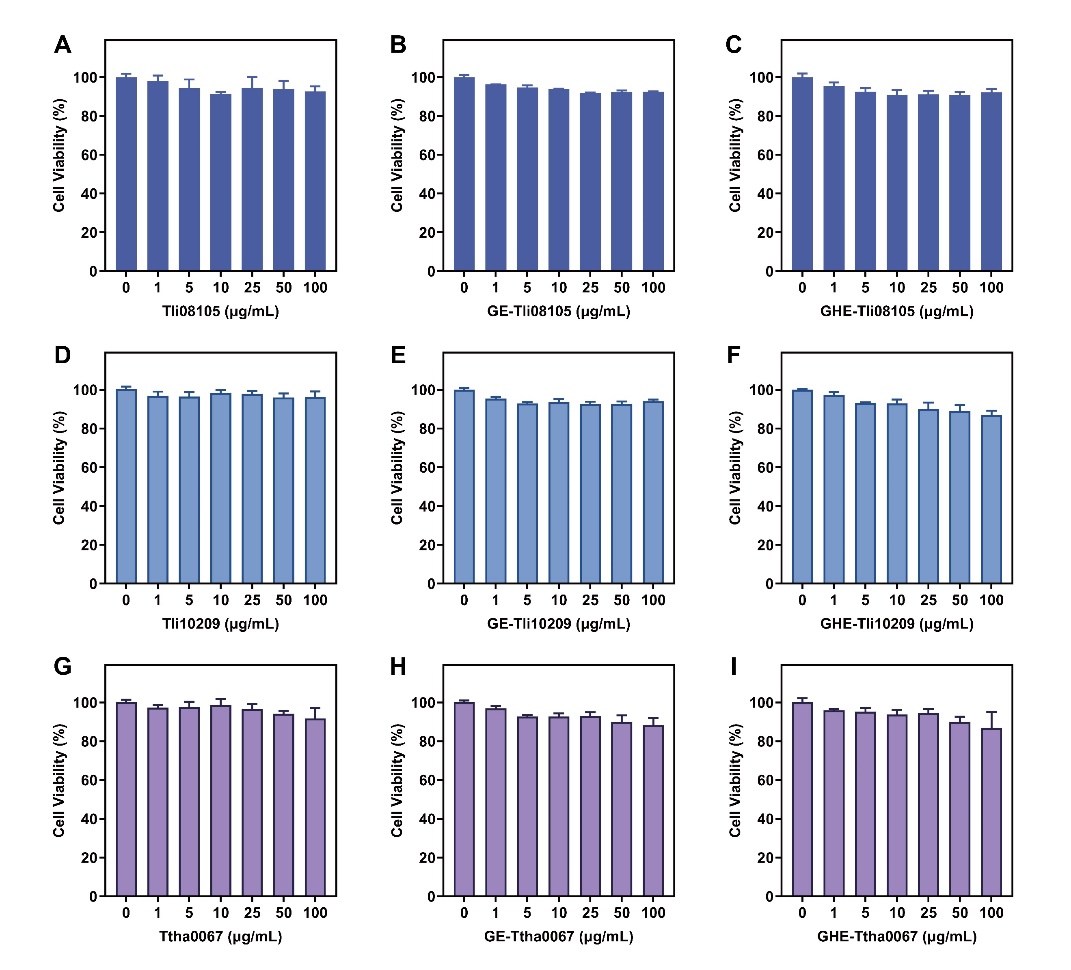


**Figure S10.** Relative viability of MCF10A cells incubated with various concentration of thermozyme/nanocatalysts.


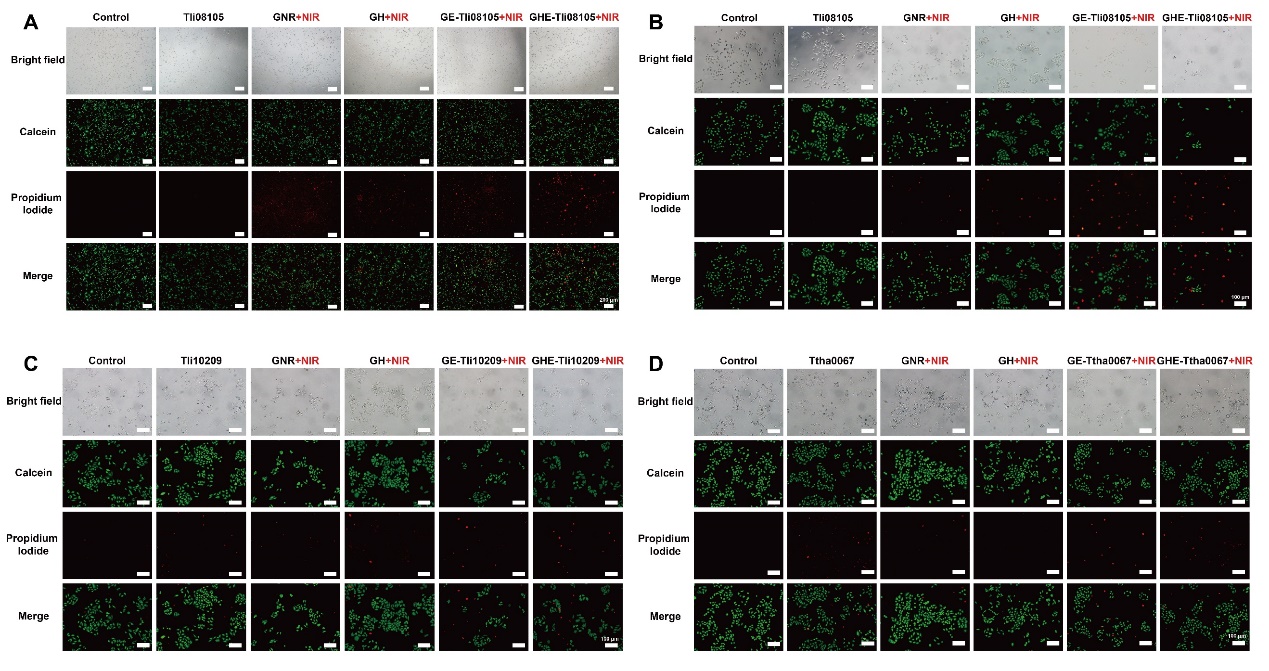


**Figure S11.** Fluorescence images of viable and dead **(A)** MDA-MB-231 and **(B-D)** MCF7 cells (stained with Calcein-AM/PI) treated with different samples. The scale bars are (A) 200 μm, and (B-D) 100 μm, respectively.


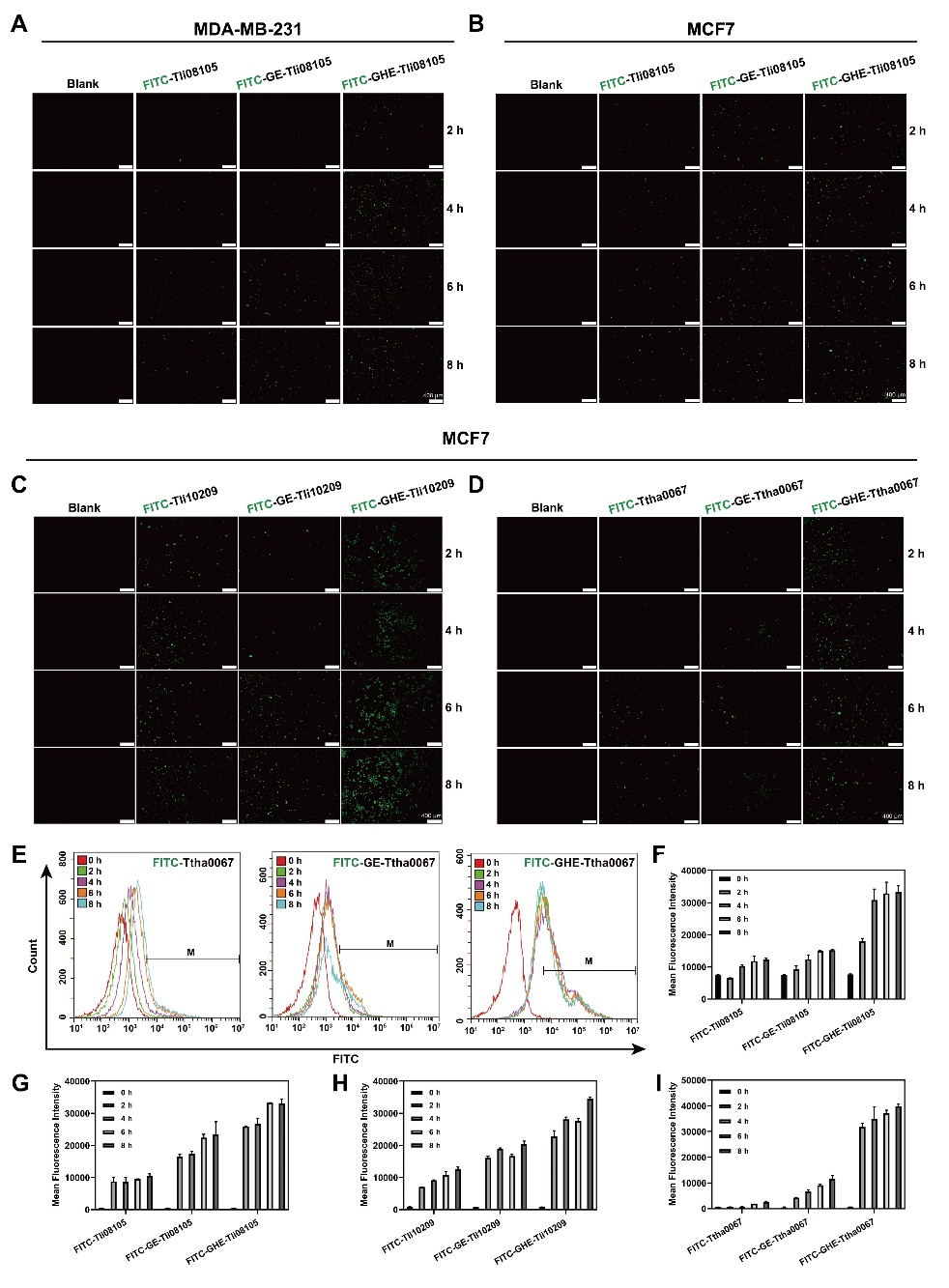


**Figure S12. *In vitro* cellular uptake assessment.** **(A-D)** Representative fluorescence images of MDA-MB-231 and MCF7 cells after co-incubation with different FITC-labeled thermozyme/GE/GHE nanocatalysts for various time at 37°C. Scale bar: 400 μm. **(E)** The corresponding quantitative fluorescence intensity of FITC-Ttha0067 with different architectures in MCF7 cells were evaluated by flow-cytometry. **(F-I)** The mean FITC-fluorescence intensity of corresponding labeled thermozyme/GE/GHE nanocatalysts co-incubated with (F) MDA-MB-231 and (G-I) MCF7 cells for different times. Data are shown as the mean ± SD (n = 3).


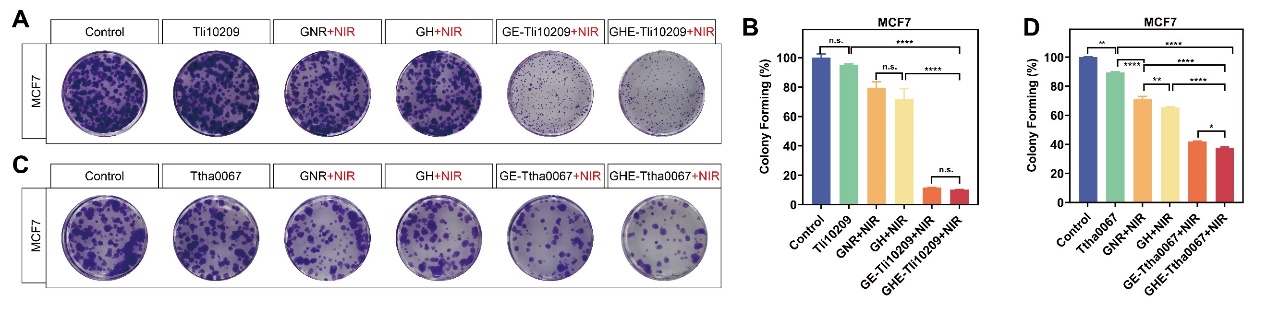


**Figure S13.** Effect of thermophilic asparaginases and NIR-triggered nanocatalysts on MCF7 cell proliferation measured by the number of colonies. Data are shown as the mean ± SD (n = 3). (n.s., not significant; ***p* < 0.01, *****p* < 0.0001)


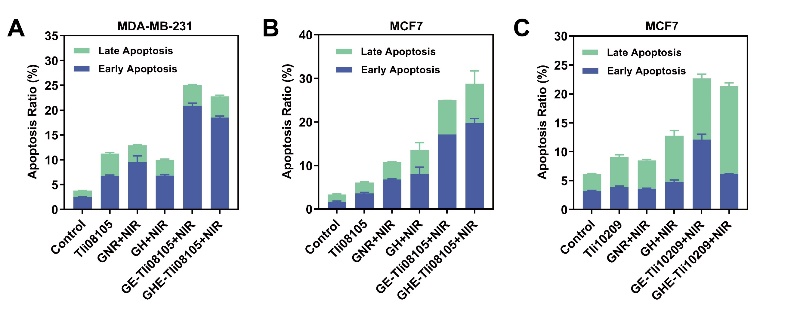


**Figure S14.** Flow cytometric quantitative analyses of Annexin V-FITC/PI co-stained **(A)** MDA-MB-231 and **(B, C)** MCF7 cells after co-incubation with different samples.


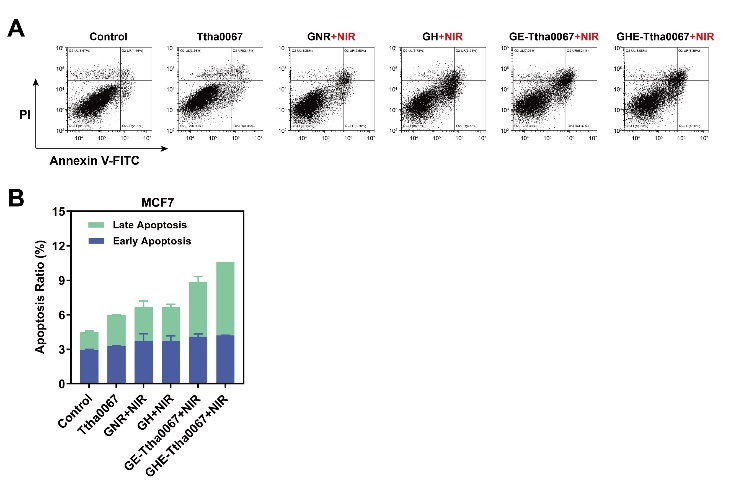


**Figure S15.** MCF7 cells were cultured with Ttha0067, GNR + NIR, GH + NIR, GE-Ttha0067 + NIR, GHE-Ttha0067 + NIR, and flow cytometry was used to measure the apoptosis ratios for each group. Data are shown as the mean ± SD (n = 3).


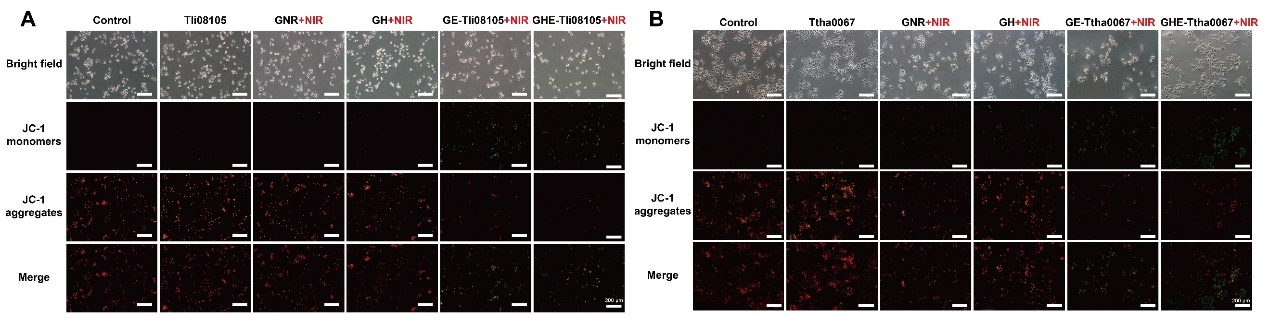


**Figure S16. (A)** Fluorescence microscopy images of JC-1-labeled MDA-MB-231 cells treated with Tli08105, GNR + NIR, GH + NIR, GE-Tli08105 + NIR, GHE-Tli08105 + NIR. **(B)** Fluorescence microscopy images of JC-1-labeled MCF7 cells treated with Ttha0067, GNR + NIR, GH + NIR, GE-Ttha0067 + NIR, GHE-Ttha0067 + NIR. Scale bar: 200 μm.


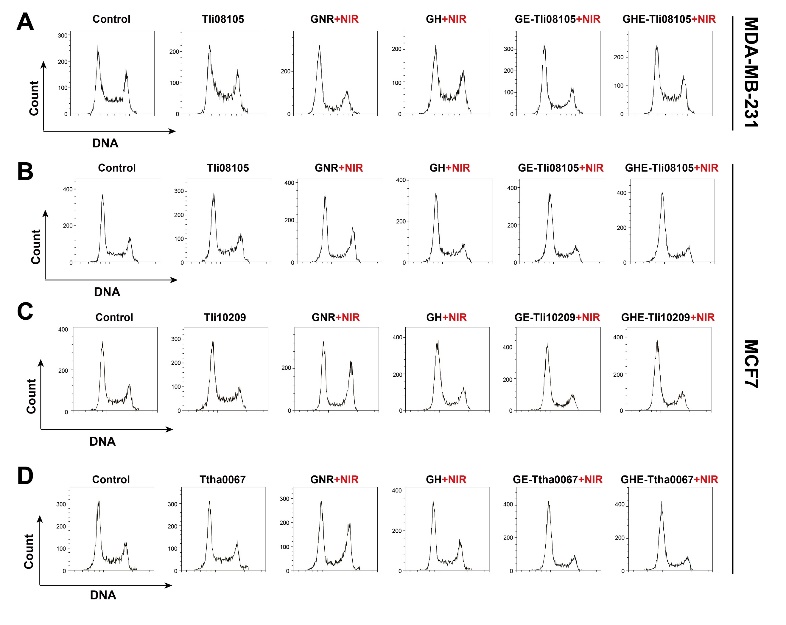


**Figure S17.** Cell cycle analysis by flow cytometry in MDA-MB-231 and MCF7 cells for the GHE-photothermal catalytic therapy with PI staining based on **(A, B)** Tli08105, **(C)** Tli10209, and **(D)** Ttha0067.


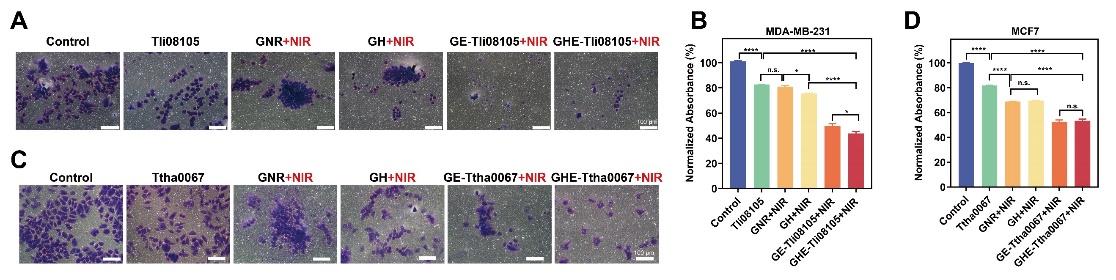


**Figure S18.** Invasion ability of **(A, B)** MDA-MB-231 and **(C, D)** MCF7 cells after different treatments. Scale bar: 100 μm. (n.s., not significant; **p* < 0.05; *****p* < 0.0001).


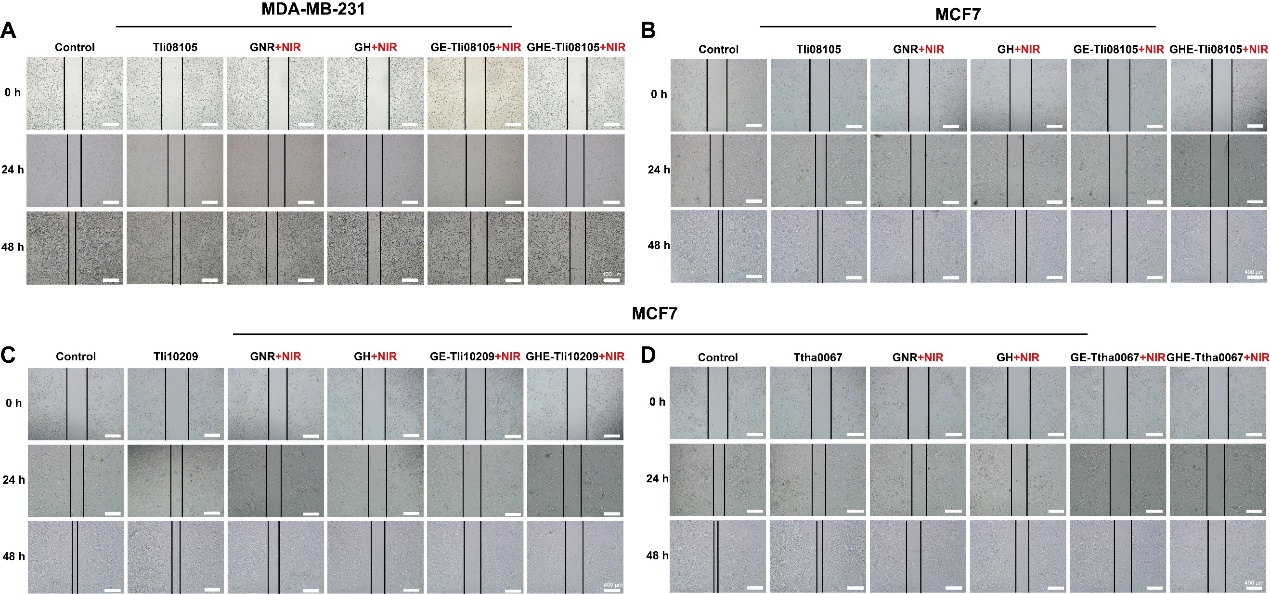


**Figure S19.** Cell migration of MDA-MB-231 and MCF7 cells after different treatments *via* wound healing assays within 48 h. Scale bar: 400 μm.


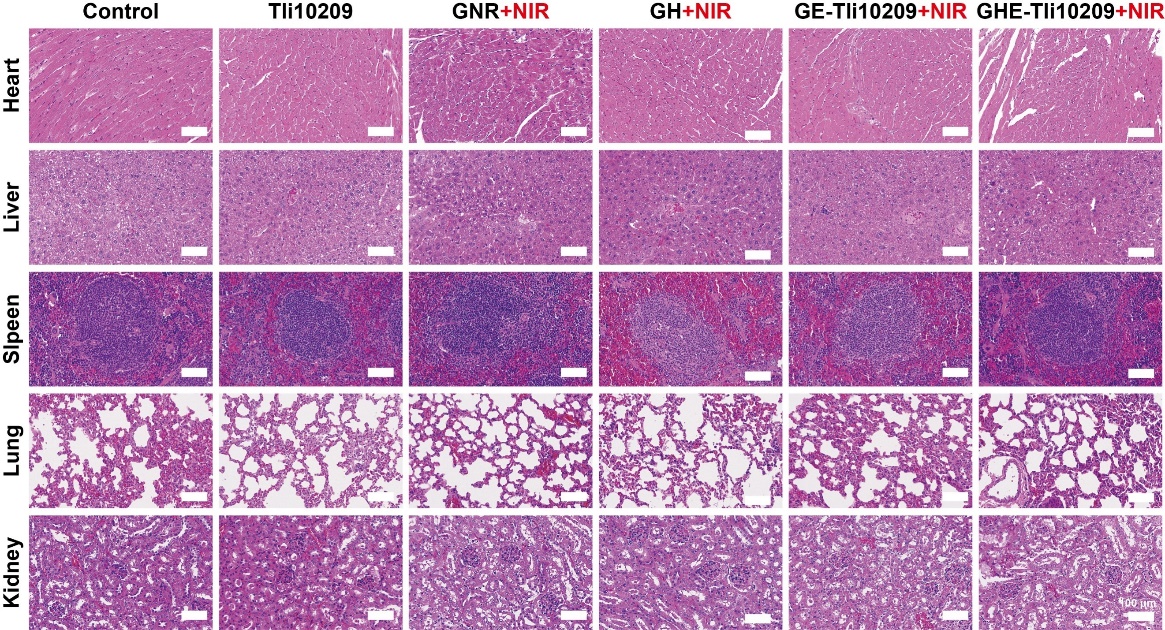


**Figure S20.** H&E staining images of major organs (heart, liver, spleen, lung, and kidney) of nude mice after various treatments for *in vivo* biosafety evaluation. Scale bar: 100 μm.


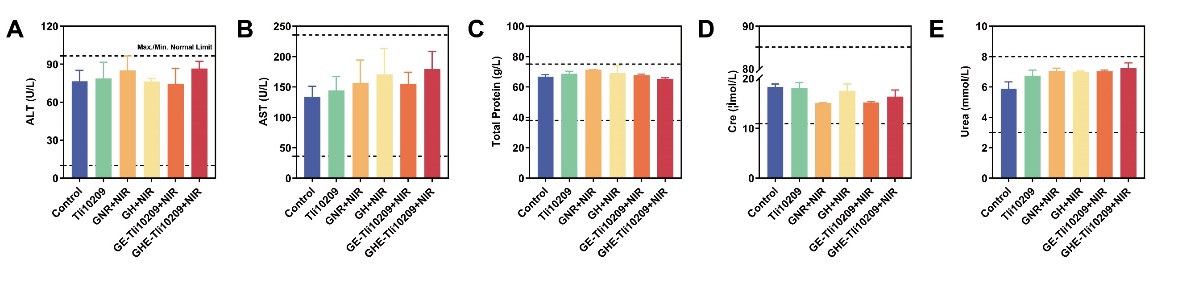


**Figure S21.** Detection of **(A)** AST, **(B)** ALT, **(C)** total protein, **(D)** Cre, and **(E)** urea content in the serum of nude mice after Tli10209-photothermal synergistic therapy. Data are shown as the mean ± SD (n = 3).
